# Detection of Polysaccharide Markers of Fungal Infections by Surface-Enhanced Raman Scattering and Machine Learning Methods

**DOI:** 10.3390/bios16090502

**Published:** 2026-09-08

**Authors:** Julia Yu. Zvyagina, Robert R. Safiullin, Andrey S. Naboko, Victor I. Polozov, Irina A. Boginskaya, Marina V. Sedova, Vadim B. Krylov, Dmitry V. Yashunsky, Dmitry A. Argunov, Nikolay E. Nifantiev, Ilya A. Ryzhikov, Alexander M. Merzlikin, Andrey N. Lagarkov

**Affiliations:** 1 Institute for Theoretical and Applied Electromagnetics RAS, 125412 Moscow, Russia; safiullinrk@gmail.com (R.R.S.); nas.webwork@gmail.com (A.S.N.); viktor.polozov@phystech.edu (V.I.P.); sedova_marina@mail.ru (M.V.S.); nanocom@yandex.ru (I.A.R.); merzlikin_a@mail.ru (A.M.M.); itae@itae.ru (A.N.L.); 2 Moscow Center for Advanced Studies, 123592 Moscow, Russia; 3 N.D. Zelinsky Institute of Organic Chemistry, Russian Academy of Sciences, 119991 Moscow, Russia; v_krylov@ioc.ac.ru (V.B.K.); yashunsky1959@yandex.ru (D.V.Y.);; 4 FMN Laboratory, Bauman Moscow State Technical University, 105005 Moscow, Russia

**Keywords:** surface-enhanced Raman scattering, polysaccharides, fungal infection, machine learning methods, partial least squares

## Abstract

In this study, we used the SERS method for the first time to measure the spectra of four polysaccharide markers of fungal infections: linear β-(1→3)- and β-(1→6)-linked D-glucans, branched mannan of *Candida albicans* and galactomannan of *Aspergillus fumigatus*. Aqueous solutions of the polysaccharides were studied in concentrations from 10 pg/mL to 100 μg/mL. The spectra were analyzed using machine learning methods: principal component analysis for data visualization and partial least squares with a ridge regularizer, which were used to construct metrics reflecting the accuracy of substance recognition relative to each other. The spectral changes with varying analyte concentration were observed and stable calibration has been achieved. Subsequent measurements of fungal polysaccharides in the presence of a physiological concentration of human serum albumin (45 mg/mL), used to model blood serum, enabled accurate analyte detection in a clinically relevant concentration range of 10 pg/mL to 100 ng/mL. In this case, the calibration dependence was calculated using the partial least squares method with the L1-regularizer. Blind testing was evaluated using a train-derived applicability-domain criterion based on the disagreement between the model prediction and an independent concentration estimate.

## 1. Introduction

According to a 2025 World Health Organization report [1], more than 6.5 million cases of invasive fungal infections and 3.8 million deaths from severe fungal diseases are registered annually worldwide. A review published in The Lancet Infectious Diseases [2] shows that aspergillosis and candidiasis are the leading causes of death among fungal infections. Each year, more than 2 million people develop invasive aspergillosis secondary to a chronic obstructive pulmonary disease, resulting in 1.8 million deaths annually (85.2% mortality). Chronic pulmonary aspergillosis accounts for 1.8 million cases annually, of which 340,000 (18.5%) are fatal. Approximately 1.5 million people develop invasive candidiasis, including candidemia, and of these patients, 995,000 (63.6%) die.

After fungal spores or cells enter the human body, they adhere to the host cells. The fungal cell wall plays an important role in recognition by the immune system [3]. It is known that the fungal cell wall consists of more than 50% glucans—polysaccharides made up of D-glucose monomers linked by glycosidic bonds. Most glucan polymers consist of β-(1→3)-linked D-glucose units (65–90%), although polysaccharides of this type containing inter-unit β-(1→6)-, β-(1→4)-, α-(1→3)-, and α-(1→4)-linkages are also found [4]. It has been shown that the cell wall of *Candida albicans* consists of 31% β-(1→3)-D-glucan (below is depicted as “β-1,3-glucan”) and 23% β-(1→6)-D-glucan (“β-1,6-glucan”) [5]. Thus, β-D-glucans in general, and β-1,3 and β-1,6 ones in particular, are especially valuable for the diagnosis of invasive fungal diseases. Whereas β-D-glucans form the basis of the rigid inner layer of the cell wall and were found in all fungi, the components of the outer layer are more species-specific markers [6]. Thus, the characteristic components of the outer layer of the *C. albicans* cell wall is mannan while of *Aspergillus fumigatus*—galactomannan [4]. Therefore the mannan and galactomannan are used as biomarkers in the diagnostic detection of *C. albicans* and *A. fumigatus*, respectively [7].

Currently, serologic testing for antibodies and antigens of various fungi is the mainstay of clinical practice. These assays include the following categories: β-1,3-glucan tests; galactomannan tests; mannan detecting and anti-mannan antibody tests [8]. The most common assay is the enzymatic β-1,3-glucan test based on the Limulus amebocyte lysate (LAL test) activation coagulation cascade [9]. According to a meta-analysis [10], the LAL test has high sensitivity for invasive mycoses, but its specificity may decrease due to possible false-positive reactions. Mannan and galactomannan which are determined in biological fluids using enzyme-linked immunosorbent assay (ELISA) [7,11] also have a significant limitation due to their limited species specificity, dependence on the localization of infection [12] and discrepancies of used antibodies and antigens [13,14,15] and the necessity of the development of new reagents of these type [16,17,18].

PCR-based assays for Candida and Aspergillus detection offer high sensitivity but rely on known species-specific probes [19,20]. Furthermore, these tests currently lack standardization and exhibit reduced sensitivity in certain patient populations. This is particularly critical for blood PCR in invasive aspergillosis under antifungal therapy, where specificity drops—likely due to colonization (in the case of Candida) or cross-reactivity with non-pathogenic species.

Mass spectrometry methods, in particular MALDI-TOF MS, represents a separate diagnostic approach and is proposed for the rapid identification of fungi, including Candida [21,22] and Aspergillus [23,24]. However, it should be noted that MALDI-TOF MS may struggle to differentiate between closely related species [25]. As with most identification methods, a pure culture is required, which limits the speed of analysis [26]. The need for highly trained personnel and expensive equipment also restricts the use of this technique in rapid clinical diagnostics.

Vibrational spectroscopy techniques have also been investigated as diagnostic tools for fungal and antigen detection. Fourier-transform infrared spectroscopy (FTIR) has proved useful for typing different Candida strains [27,28] and for differentiating toxigenic and non-toxigenic *Aspergillus* isolates of agricultural importance [29]. In the described studies, fungal cultures were obtained for analysis after growth periods ranging from 1 to 14 days. Due to the measurement procedure specifics, infrared spectroscopy is characterized by relatively low sensitivity to individual cell wall components, especially at low concentrations. Another significant limitation is the strong absorption of infrared radiation by water, which complicates the analysis of aqueous solutions and biological fluids.

Raman spectroscopy is particularly well suited to the analysis of biological samples owing to several advantages, including the ability to obtain detailed information on the molecular groups structure, minimal requirements for sample preparation, and low sensitivity to the presence of water. In the late twentieth century, Edwards et al. [30] demonstrated the applicability of Raman spectroscopy for studying the fungi *Aguricus bisporus*, *Mortierella*, and *Mucor* and identified characteristic Raman bands corresponding to cell wall components. Subsequently, De Gussem et al. [31] showed, using spores of the genus Lactarius as a model, that the recorded signals predominantly originate from cell wall polysaccharides, including glucans. However, conventional Raman spectroscopy has a number of limitations. Because the method is based on inelastic light scattering, the signal intensity is relatively low, which limits the analysis sensitivity. This significantly restricts its applicability to the complex biological object analysis without the use of additional signal amplification approaches.

Surface enhanced Raman scattering (SERS) is a promising tool for detecting and identifying pathogenic microorganisms [32,33,34]. SERS amplifies the Raman signal of molecules adsorbed on nanostructured metal surfaces (gold, silver), enabling analyte spectra to be recorded at extremely low concentrations [35]. This approach provides a molecular “spectral fingerprint” reflecting the structure of the analyzed compounds, requires minimal sample volume, and significantly reduces analysis time. The choice of SERS substrate architecture depends strongly on the target application. Complex hybrid structures incorporating perovskites have shown high sensitivity for veterinary drug detection in food matrices [36], and bismuth-containing composites have been proposed for pesticide and veterinary drug screening [37]. In the context of clinical diagnostics, planar nanostructured silver thin-film substrates [38,39] present a favorable balance of enhancement factor, spatial homogeneity, and long-term stability, as demonstrated previously for the SERS-based quantification of angiotensin-converting enzyme in serum samples. One of the pioneering studies by E. Witkowska et al. [40] demonstrated that SERS combined with Principal Component Analysis (PCA) successfully differentiates the spectra of four fungal pathogens: *Trichophyton rubrum*, *C. krusei*, *Scopulariopsis brumptii*, and *A. flavus*. The authors used pure fungal cultures grown on agar medium for 3 weeks. Similar studies differentiated species [41] and strains [42,43] of some fungi, thereby expanding the range of applications of SERS in the diagnosis of fungal infections. In addition to the direct detection of fungal microorganisms, SERS can be used in conjunction with other diagnostic methods. For example, to avoid false-positive and false-negative ELISA results, the combined use of ELISA and SERS has been proposed for galactomannan identification [44]. The potential for combining SERS with multiplex Polymerase Chain Reaction (PCR) testing to detect different strains of *Candida* and *Aspergillus* has also been demonstrated [45]. A recent review [32,46,47,48] on the use of SERS for detecting fungi, bacteria, and viruses highlights the rapidly expanding functionality of this method in medical diagnostics. Previously, our group demonstrated the feasibility of detecting structures containing the β-D-galactofuranoside (Galf) residues using SERS [49]. Galf is a characteristic component of a variety of fungal and bacterial glycans including *Aspergillus* galactomannan and is normally absent from the human body, making it a highly specific marker of certain infection agents. The detection of characteristic vibrational bands of Galf-residues confirms the potential of SERS for identifying species-specific fungal structures. Quantitative determination of analytes can also be achieved using SERS combined with machine learning methods. The use of PCA, Partial Least Squares (PLS), Support Vector Machine (SVM), and a number of other methods has previously been demonstrated for the determination of glycated albumin in model plasma [50,51], as well as the quantitative determination of proteins using dielectrophoretic precipitation [52]. This suggests that similar approaches can be implemented for a wider range of biologically significant analytes, including oligo- and polysaccharides.

This study presents the first SERS spectral investigation of β-1,3- and β-1,6-glucans, as well as mannan of *C. albicans* and galactomannan of *A. fumigatus*. Aqueous solutions of the analytes studied over a concentration range of 10 pg/mL to 100 μg/mL. Measurements were also performed in the presence of a physiological concentration of human serum albumin (45 mg/mL), used to model blood serum, with analyte concentrations ranging from 10 pg/mL to 100 ng/mL. Concentrations from several tens of pg/mL to several hundred of ng/mL are considered clinically significant for these fungal polysaccharides. Because of the structural similarity of the molecules, multivariate statistical and machine learning methods were used for a more accurate and rapid analysis of the spectra. For dimensionality reduction and visualization, PCA was applied, taking into account the contribution of the first two components. Then, using PLS analysis, classification quality metrics were calculated, which reflect the recognition accuracy of the studied substances. For the first time, concentration-dependent dynamics of spectral profiles with decreasing concentration has been demonstrated for aqueous solutions. Concentration calibration with blind testing was performed for the model solutions.

## 2. Materials and Methods

### 2.1. Research Object

**Figure 1:**
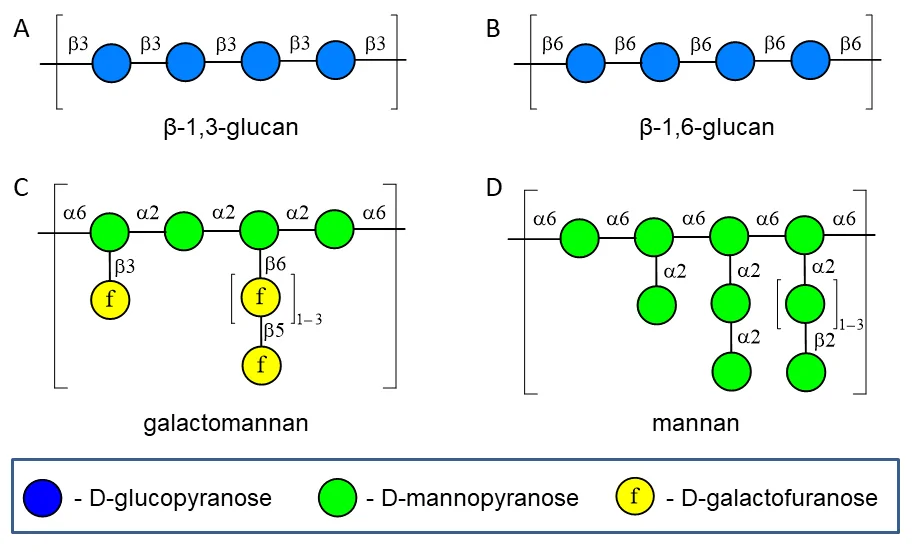
Tentative structure of fungal polysaccharides: (**A**) β-1,3-glucan; (**B**) β-1,6-glucan; (**C**) galactomannan; (**D**) mannan. The carbohydrate sequences are represented according to symbol carbohydrate nomenclature (SNFG) [60].

The tentative structures of key studied polysaccharides are present at Figure 1. The common fungal polysaccharide β-1,3-glucan was isolated by extraction from *Saccharomyces cerevisiae* according to the procedure described in [53]. β-1,6-Glucan and mannan were isolated from a culture of *C. albicans* following protocols published in [5] and [54], respectively. Galactomannan was isolated by extraction from a culture of *A. fumigatus* according to the standard protocol described [55,56]. The structural identity of the isolated polysaccharides was confirmed by their NMR spectroscopic data which were consistent with described data for β-1,3-glucan [57]; β-1,6-glucan [5]; mannan [58]; galactomannan [56,59].

### 2.2. SERS Substrate Fabrication and Characterisation

SERS substrates were fabricated according to the protocol described in [61] and consist of a thin nanostructured self-organized silver film deposited on glass. The deposition was carried out in an automated ultrahigh-vacuum electron-beam evaporation system STE EB71M (SemiTeq JSC, Saint Petersburg, Russia). A high-purity silver source (>99.99%, “Special Alloys Processing Plant”, Moscow, Russia) in the form of granules up to 3 mm in size was used for evaporation. Silver was evaporated using an 8 kW electron gun (Telemark, Battle Ground, WA, USA) with an accelerating voltage of 10 kV. The ultimate pressure in the deposition chamber reached 2 × 10^−8^ Torr (3 × 10^−6^ Pa). The film thickness was monitored with a quartz crystal microbalance (Inficon, Bad Ragaz, Switzerland) and set to 200 nm. Silver was deposited onto standard microscope slides (Minilab LLC, Dyatkovo, Russia). Prior to deposition, the glass slides were mechanically cleaned with surfactants, rinsed in ethanol and then in deionized water using a cascade bath, and subsequently dried in a laminar flow hood. SERS substrates were fabricated in three independent batches (three separate electron-beam evaporation runs), with 12 substrates per batch. Substrate homogeneity was assessed in terms of morphological, spectral, and temporal stability parameters.

The morphological homogeneity of the fabricated SERS substrates was investigated by atomic force microscopy (AFM) using a Solver Pro microscope (NT-MDT, Zelenograd, Russia) in tapping mode. For measurements, substrates were randomly selected from different batches. AFM image analysis and processing were performed using Gwyddion software version 2.60 (CMI, Brno, Czech Republic). Surface morphology was characterized in compliance with ISO 21920-2:2021 (Geometrical Product Specifications—Surface Texture), ensuring standardized quantification of amplitude, spatial, and statistical roughness parameters [62]. This unified metrological framework enabled direct quantitative comparison of morphological features across independently fabricated substrate batches.

The spectral homogeneity of the substrates was assessed by comparing the SERS spectra of the investigated analytes acquired on substrates from different fabrication batches. For each sample, three independent 3 μL droplets were deposited on spatially separated areas of the substrates. For each droplet, 15–20 spectra were recorded at randomly selected positions distributed over the entire dried deposit, resulting in a total of 50 spectra per sample. All spectra were acquired under identical experimental conditions, using an excitation wavelength of 785 nm and a laser power of 25 mW. This approach provided spatial sampling of the SERS response and allowed potential signal heterogeneity both within individual dried deposits and between independent sample depositions to be taken into account. For a correct comparison, the original spectra were subjected to minor pre-processing: cutting in the significant spectral range of 300–1800 cm^−1^ and subtraction of the baseline using the concave rubber band correction algorithm (iterations *n* = 10; baseline points *n* = 64).

The spectral reproducibility was quantitatively evaluated using the relative standard deviation (RSD) of the intensity of characteristic spectral bands:

$$RSD=\frac{SD}{\bar{I}}\times 100\%$$

where SD is the standard deviation of the intensity of the respective band and $\bar{I}$ is its mean intensity. The resulting RSD values were compared for spectra acquired on substrates from different fabrication batches, allowing the batch-to-batch reproducibility of the SERS response to be assessed.

### 2.3. Analyte Preparation and Application

For SERS measurements, solutions of the polysaccharide markers were prepared in deionized water (Milli-Q, Merck, Darmstadt, Germany) across a concentration range from 100 µg/mL to 10 pg/mL with tenfold dilution steps. To establish calibration curves, model solutions based on human serum albumin (HSA) were prepared to simulate human blood serum. For this purpose, HSA solution was prepared in deionized water at a physiological concentration of 45 mg/mL, and stock solutions of the target analytes were added to obtain spiked solutions with a constant HSA concentration and defined analyte concentrations from 100 ng/mL to 10 pg/mL. Blind testing of the calibrations was performed using spiked solutions with analyte concentrations of 150 and 500 pg/mL.

Prior to SERS measurements, all samples were deposited onto SERS substrates as 3 µL droplets and then dried in a temperature-controlled chamber at 25 °C. SERS spectra of the analytes were recorded from the dried precipitate region.

### 2.4. Spectra Measurements

SERS spectra were measured on an Alpha 300R confocal Raman microscope (WITec, Ulm, Germany) using a Zeiss Epiplan-Neofluar 50×/0.8 objective (Carl Zeiss AG, Oberkochen, Germany). The excitation laser wavelength was 785 nm, and the laser power was 25 mW. The spectra were acquired in single-spectrum mode with an integration time of 15 s and a single accumulation.

For the aqueous analyte solutions, 50 spectra were recorded per sample. A sample is considered to be one substance in one concentration. For the spiked HSA solutions, 50 spectra were also acquired per sample. For the blind test samples, 50 spectra were measured per sample.

### 2.5. Pre-Processing of Spectral Data

The SERS spectrum of each measurement was represented as a feature vector X, whose components corresponded to intensities at fixed Raman shifts. This representation transforms spectral analysis into a statistical learning problem in a multidimensional feature space and enables a unified framework for (i) comparing spectra, (ii) classifying analytes, and (iii) constructing calibration curves.

Spectral preprocessing was aimed at suppressing contributions that did not carry analytical information about the studied polysaccharide and at reducing intermeasurement variability inherent to SERS, including hot-spot heterogeneity, background contributions from the substrate and matrix, and changes in the overall intensity scale during spectral acquisition. The spectra were truncated to the informative spectral range of 260–1750 cm^−1^. Baseline correction was then applied, and the spectra were brought to a comparable scale by standard normal variate (SNV) normalization, allowing the analysis to focus primarily on band changes rather than random fluctuations of the fluorescence background. After preprocessing, all spectra were represented on a common Raman-shift range and assembled into the feature matrix X for subsequent calculations.

Outlier removal was performed at the level of entire spectra using the Isolation Forest algorithm [63]. This method identifies spectra that differ substantially from the main body of data in the multidimensional feature space. Spectra classified as outliers at a specified contamination level were excluded from further analysis. This procedure eliminates measurements with anomalous behavior caused, for example, by signal instability, acquisition artifacts, or poor spectral quality.

Baseline correction was performed using asymmetric penalized least-squares smoothing (asPLS) [64], in which a background component was estimated for each spectrum and subtracted from the original signal. This approach effectively removes slowly varying background associated with fluorescence and substrate contributions without distorting analytically significant bands.

For quantitative concentration determination, the target variable was defined on a logarithmic scale:

y = log_10_(C + ε),

where C is the analyte concentration and ε is a small offset introduced to avoid taking the logarithm of zero. The logarithmic scale is justified by the broad concentration range used in the experiment: under this parameterization, model error is interpreted as an error in the order of magnitude of concentration and becomes more comparable across concentrations.

### 2.6. General Problem Formulation and Split Design

The analysis was performed in two complementary settings.

(1a) Classification: comparison of analytes at a fixed concentration. Given the spectral vector X, the analyte class (β-1,3-Glc, β-1,6-Glc, Ag. C.a., or Ag. A.f.) was determined within a set of measurements corresponding to a single concentration. The aim of this stage was to assess the spectral separability of the substances and to trace how it changes with concentration, including changes associated with reduced statistical error and the increasing influence of background and SERS spectral variability.

(1b) Classification: comparison of different concentrations of the same substance. Given the spectral vector X, the concentration class was determined within a set of measurements corresponding to a single substance (concentrations of β-1,3-Glc, concentrations of β-1,6-Glc, concentrations of Ag. C.a., or concentrations of Ag. A.f.). The aim of this stage was to assess the spectral separability of different concentrations of the same substance and to trace the concentration-dependent evolution of spectral profiles.

(2) Regression: calibration in the HSA matrix. Given a spectrum X, y = log_10_(C + ε) was predicted for HSA-based spiked solutions. Generalization performance was evaluated using splits that simulated blind application of the calibration: training and test data were formed from independent measurement sets.

This design reflects the practical scenario of model application, in which calibration is built on a limited set of training data and then used to analyze new, previously unseen spectra. In this setting, the model must not only describe the training data accurately but also transfer correctly to new measurements by relying on the learned relationship between spectral profile and concentration.

Splitting the data into independent sets makes it possible to assess how well the model preserves its predictive performance on new data and how robust it is to variations that arise during spectral acquisition. Thus, the analysis addresses not only approximation but also calibration transfer, which is a key requirement for practical application of the method.

#### 2.6.1. Dimensionality Reduction and Visual Inspection (PCA)

Principal component analysis (PCA) was used for initial analysis of the data structure and visualization of spectral distributions. PCA was applied to the spectral feature matrix X, and projections onto the first two principal components (PC1–PC2) were analyzed. This step served as a quality check for class clustering, overlap between point clouds, and possible artifacts or shifts between substances at different concentrations. For additional interpretation of the PCA score plots, the corresponding PC1 and PC2 loading spectra are provided in the Supplementary Materials. Supplementary Figure S1 presents the loading spectra for the fixed-concentration PCA models, whereas Supplementary Figure S2 presents the loading spectra for the concentration-series PCA models. In both figures, the loading curves are shown together with the corresponding mean SERS spectra to facilitate interpretation of the Raman-shift regions contributing to the principal-component variance.

#### 2.6.2. Classification of Spectra at Fixed Concentration or Fixed Analyte

**Linear classification models** were used for quantitative assessment of analyte separability: partial least squares regression and ridge regularization. Their use in spectral diagnostics is justified by the fact that, after appropriate preprocessing, a linear boundary in feature space is often sufficient, while the model coefficients retain a direct physical interpretation as the contribution of each spectral coordinate to the decision. The confusion matrix and classification accuracy were used as quality metrics; the analysis was performed separately for each dataset corresponding to a fixed concentration.

**Data splitting.** Within each concentration-specific dataset, spectra were randomly divided into training and test subsets while preserving class proportions (stratification), ensuring correct class comparison even when class sizes differed.

**Confusion matrix**. The confusion matrix was constructed from the true and predicted class labels in the test subset. Diagonal elements correspond to the number of correct predictions, whereas off-diagonal elements indicate specific pairs of classes confused by the model. This format makes it possible to interpret not only the overall accuracy but also the nature of the errors, such as systematic confusion between two structurally similar analytes with shared vibrational bands.

#### 2.6.3. Identification of Spectral Regions Responsible for Class Separation

To interpret the classification results, the coefficients of the linear models were used as estimates of feature contributions. Because each feature corresponds to the intensity at a specific Raman shift, classifier coefficients can be directly mapped to spectral regions and interpreted as indicators of diagnostically significant bands.

In practice, spectral bands with the largest absolute coefficient values, corresponding to the most discriminative features, were selected and compared with band positions in the averaged spectra. This approach makes it possible to move from formal classification quality to physicochemical interpretation by identifying which spectral regions ensure compound separability and how well these regions are preserved as concentration or substance varies. The approach is consistent with the general logic of machine-learning-integrated surface-enhanced spectroscopy, in which interpretable models link classification results to spectral markers.

**Plot method**: loadings and selected bands. In the corresponding figures, averaged class spectra are shown as solid lines, whereas the positions of the most significant spectral coordinates are indicated by dashed vertical lines. Color coding is used to match each class or concentration with its spectrum and selected coordinates.

#### 2.6.4. Calibration Curves in HSA

Concentration calibration in the HSA model matrix, which simulates serum, was formulated as a regression problem: given the spectral vector X, the model predicted

y = log_10_(C + ε).

Partial least squares with L1 regularization (PLS-L1) was used as the regression model. This choice was motivated by the high dimensionality and strong correlation of spectral features characteristic of SERS data. PLS projects the original feature space into a latent space that maximizes covariance with the target variable, whereas L1 regularization promotes sparsity of the solution and suppresses irrelevant and noisy spectral coordinates. This approach increases model robustness to spectral variability and improves generalization when constructing calibration curves.

Before training, the features were standardized by centering and scaling to bring all spectral coordinates to a comparable scale and stabilize model fitting. Model tuning was performed on the training subset using internal cross-validation; final quality assessment was carried out on an independent test series that was not used for parameter selection.

**Calibration plot.** Calibration was visualized in coordinates of true y versus predicted y with the diagonal of ideal agreement added. The same plot shows training-sample points, used to verify model consistency with the training data, and test-sample points, used as the primary estimate of generalization. In addition, a confidence band around the diagonal was plotted from the variance of residuals in the training set, enabling visual assessment of whether test predictions lie within the typical model error.

#### 2.6.5. Train-Derived Spectral-Trajectory Criterion for Selecting Representative Blind Spectra

To define an applicability domain without using the reference concentration of an unknown specimen, an independent concentration estimate was obtained from the spectral position of each spectrum relative to the training data. The baseline-corrected and SNV-normalized Raman variables were centered using the training set only, and PCA was fitted separately for each polysaccharide using the training spectra.

Let *c_k* denotes the mean PCA score vector (centroid) for the *k*-th training concentration level *y_k*, where the training levels are ordered from lower to higher concentration. Thus, *k* refers only to a known concentration in the training set, and *k* + 1 denotes the next adjacent training concentration; neither index contains information about the measured concentration of a blind sample.

For each blind spectrum *i*, its PCA score vector was projected onto the nearest segment connecting two adjacent training centroids *c_k* and *c*_{*k* + 1}. The normalized position of the projection along this segment was denoted by *t_i*, with *t_i* = 0 at *c_k*, *t_i* = 1 at *c*_{*k* + 1}, and 0 < *t_i* < 1 for intermediate positions. Both the segment (*k*, *k* + 1) and *t_i* were determined exclusively from the spectral coordinates of the blind spectrum and the training trajectory.

*ŷtrajectory,i* = *y*_*k* + *t*_*i*(*y*_{*k* + 1} − *y*_*k*),   0 ≤ *t*_*i* ≤ 1.

In parallel, the PLS-L1 model produced the concentration prediction *ŷPLS-L1,i*. The applicability score was defined as the absolute disagreement between the PLS-L1 prediction and the independently obtained trajectory-based estimate:

*a*_*i* = |*ŷPLS-L1,i* − *ŷtrajectory,i*|.

A small *a_i* indicates that the regression prediction is consistent with the spectral position of the sample relative to the training concentration trajectory, whereas a large *a_i* indicates disagreement between the two estimates. For the training spectra, the same score was calculated using leave-one-out concentration centroids, so that the evaluated spectrum did not contribute to the centroid used for its trajectory estimate. The acceptance threshold was fixed at the 95th percentile of the resulting training-score distribution, and a blind spectrum was accepted when *a_i* did not exceed this threshold. The measured concentrations of the blind samples were not used to select the segment, calculate *t_i* or *a_i*, define the threshold, or establish the acceptance mask; they were introduced only after the mask had been fixed to construct distributions of the train-derived spectral-trajectory applicability score for the PLS-L1 calibration and calculate validation metrics. Because the individual spectra are technical measurements within independently prepared blind specimens, specimen-level predictions were calculated as the median of the accepted spectrum-level predictions.

#### 2.6.6. Spectral Feature Importance Assessment in Regression

For the regression model, whose raw coefficients were not interpreted as direct weights of individual spectral coordinates, feature importance was assessed by permutation importance: each spectral coordinate was randomly permuted in turn, and the resulting deterioration in prediction quality on the training set was measured. Coordinates whose permutation caused the greatest deterioration were interpreted as the spectral regions most important for calibration.

## 3. Results and Discussion

### 3.1. SERS Substrate Characterization

**Figure 2:**
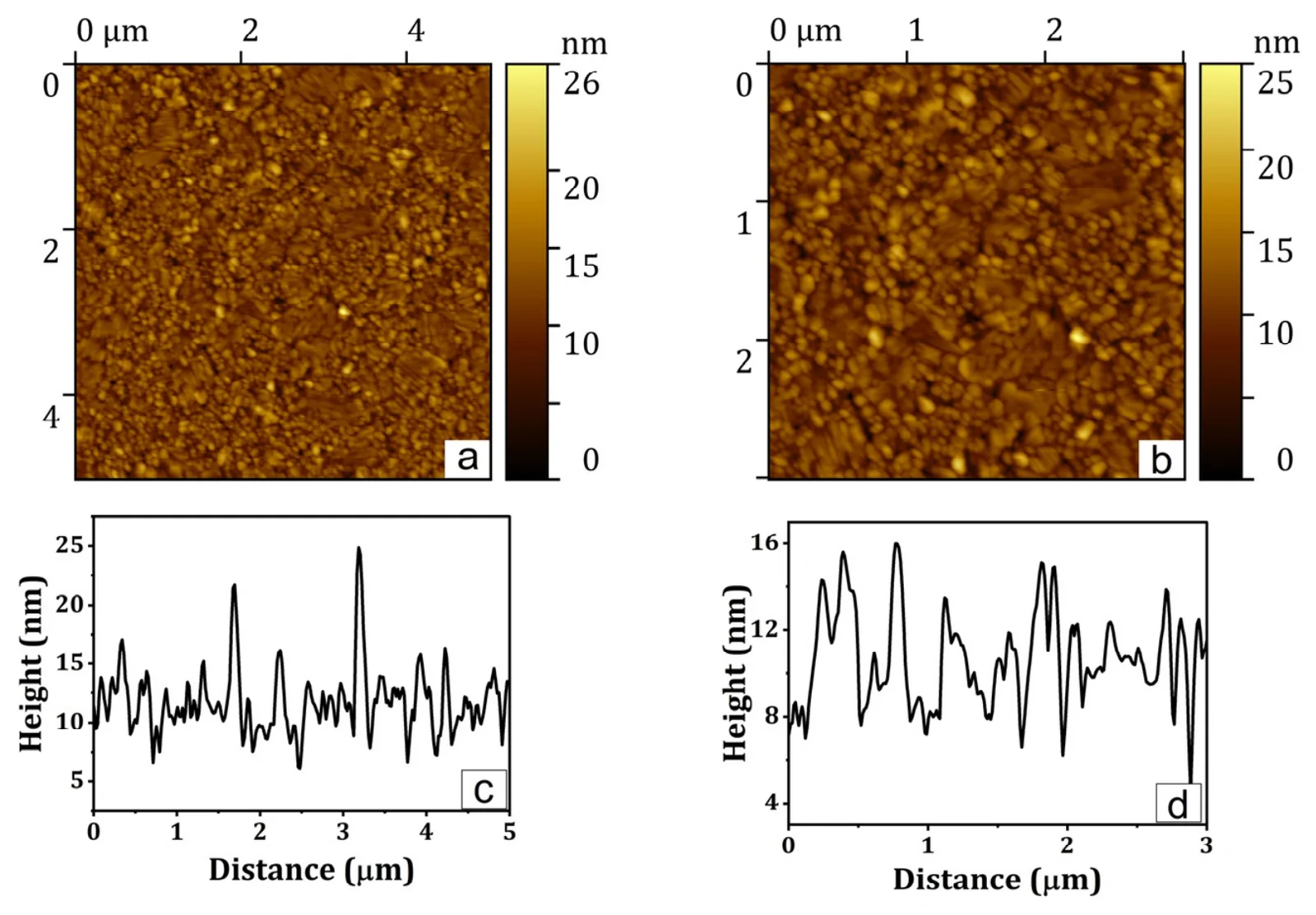
Typical AFM image of the SERS substrate surface areas with dimensions: (**a**) 5 × 5 μm; (**b**) 3 × 3 μm; cross-section on the corresponding surface area (**c**) 5 × 5 μm; (**d**) 3 × 3 μm.

Based on the AFM images of the SERS substrate surface morphologies presented in Figure 2a,b, the substrates represent polycrystalline self-organized nanostructured surfaces. Nanoscale irregularities are clearly observed in the surface cross-sectional profiles (Figure 2c,d).

AFM measurements revealed a root-mean-square (RMS) surface roughness of R_q_ = 2.2 ± 0.5 nm. This value indicates moderate nanoscale roughness and suggests that the metal film is continuous while retaining a distinct nanoscale texture. The parameter R_3z_ = 9.8 ± 0.9 nm, defined as the mean height of the three highest peaks and deepest valleys, reflects the amplitude of the most pronounced topographic features. The substantially higher R_3z_ relative to R_q_ points to the presence of individual taller nanoscale peaks or deeper valleys, which can serve as localized electromagnetic field enhancement sites and promote the formation of “hot spots” [65,66]. The profile kurtosis R_ku_ = 4 ± 0.11 exceeds 3, the value expected for a normal (Gaussian) height distribution, implying the presence of distinct isolated peaks. The positive skewness R_sk_ = 0.21 ± 0.05 indicates a predominance of protruding topographic features over valleys. The autocorrelation length λ_a_ = 180 ± 15 nm characterizes the lateral scale of surface inhomogeneities and can be interpreted as the mean spacing between neighboring morphological features. This value indicates the formation of a quasi-periodic nanostructure with a characteristic period on the order of hundreds of nanometers. Thus, the combination of parameters R_q_, R_3z_, R_ku_, R_sk_, and λ_a_ demonstrates the formation of a nanostructured surface with moderate roughness, pronounced local peaks, and a characteristic lateral scale of approximately 180 nm. The observed morphology can facilitate electromagnetic field localization and, consequently, the onset of the SERS effect [67]. The RSD of all measured morphological parameters (R_q_, R_3z_, R_ku_, R_sk_, λ_a_) across substrate areas from three independent batches did not exceed 25% of the mean value. Since the AFM measurements were performed at spatially separated locations on substrates originating from different fabrication batches, the observed variability indicates a high degree of morphological reproducibility both within the individual substrate and between independent substrate batches.

**Figure 3:**
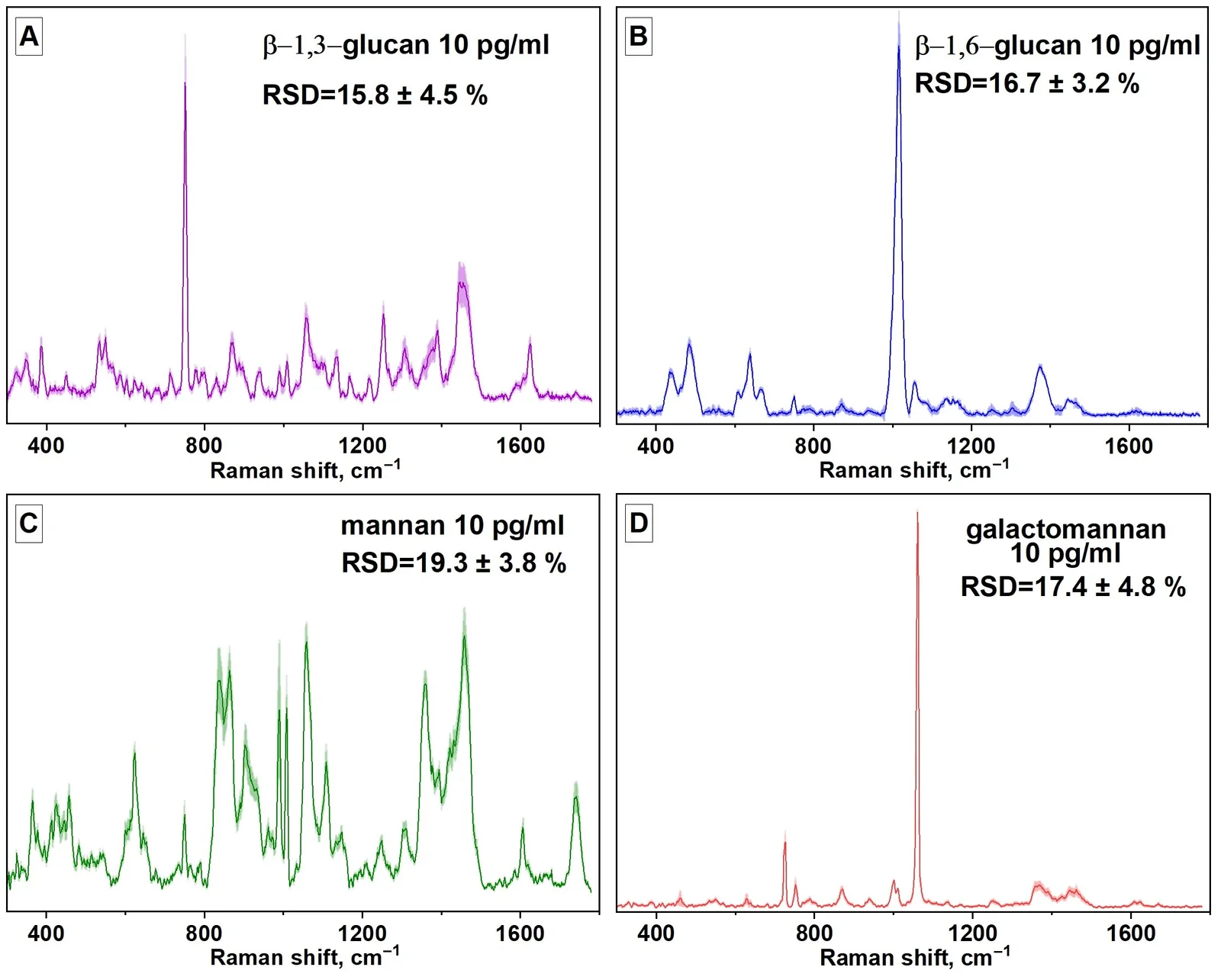
Average SERS spectra of (**A**) β-1,3-glucan, (**B**) β-1,6-glucan, (**C**) mannan and (**D**) galactomannan in the concentration of 10 pg/mL recorded from different substrates from three independent batches. Solid lines represent the mean spectra (*n* = 50 individual measurements per analyte, 15–20 per batch); shaded bands indicate the standard deviation. The RSD of the characteristic band intensities is indicated for each analyte.

To evaluate the spectral reproducibility of the SERS substrates, we compared the SERS spectra of the four analytes (β-1,3-glucan, β-1,6-glucan, mannan from *C. albicans*, and galactomannan from *A. fumigatus*) deposited on substrates from different fabrication batches. Figure 3 shows the averaged SERS spectra of the four analytes recorded on substrates from three independent batches.

Figure 3 presents the averaged spectra (solid lines) with the corresponding standard deviation bands (shaded areas). RSD of the intensities of the most intense characteristic bands was 15.8 ± 4.5% for β-1,3-glucan, 16.7 ± 3.2% for β-1,6-glucan, 19.3 ± 3.8% for mannan, and 17.4 ± 4.8% for galactomannan. These values, all below 25%, confirm acceptable spectral reproducibility of the fabricated SERS substrates and support their suitability for quantitative analysis.

**Figure 4:**
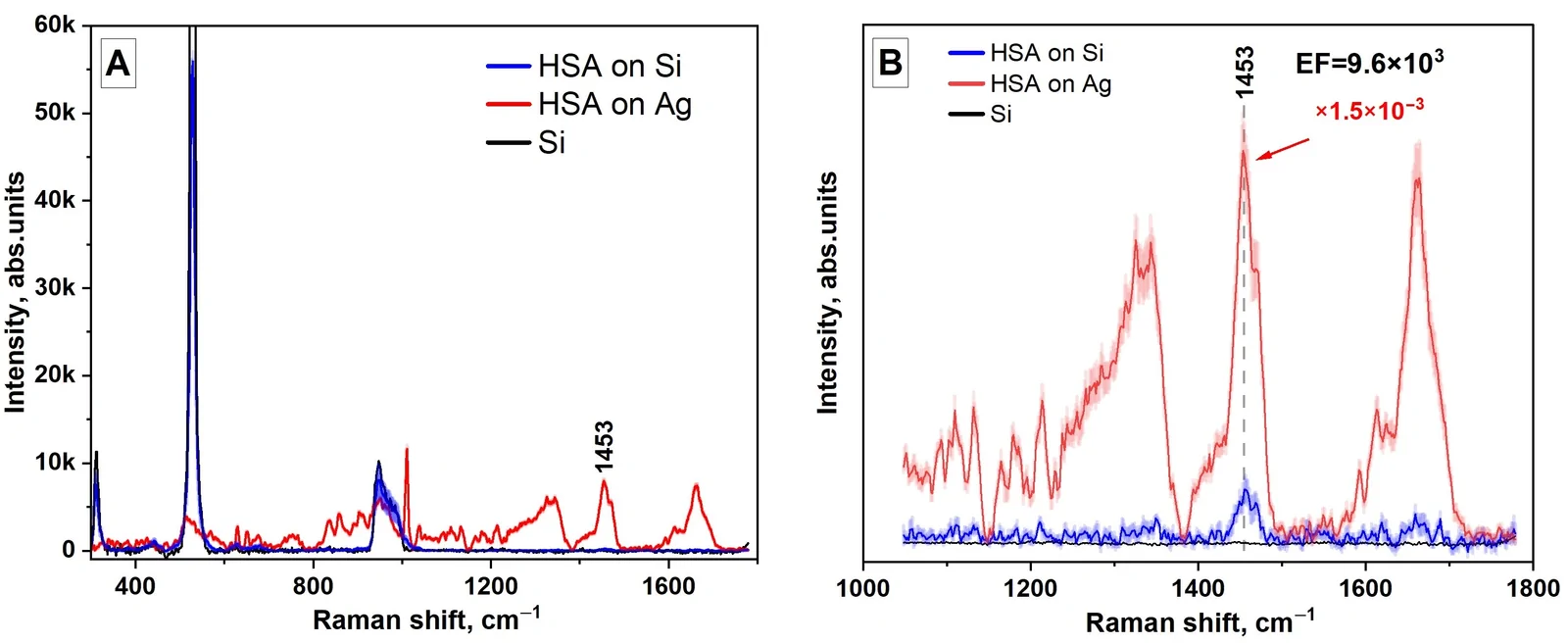
Raman and SERS spectra of HSA 1 mg/mL obtained under identical conditions on a silicon substrate and the SERS-active silver film, presented in the range (**A**) 300–1800 cm^−1^ and (**B**) 1050–1770 cm^−1^. Solid lines represent the mean spectra (*n* = 50 individual measurements per analyte, 15–20 per batch in case of SERS substrate); shaded bands indicate the standard deviation.

To determine the SERS enhancement factor (EF) of the fabricated silver substrates, a control experiment was performed in which the Raman spectra of HSA at a concentration of 1 mg/mL acquired on the SERS-active silver film were compared with those obtained on a bare silicon wafer lacking plasmonic enhancement. At concentrations below 1 mg/mL, HSA signals were indistinguishable from noise on the Si substrate under the employed measurement conditions (785 nm, 25 mW, 15 s integration, 50×/0.8 objective). The corresponding spectra are shown in Figure 4A. Because the intrinsic Raman bands of silicon overlap with the HSA spectrum below 1000 cm^−1^, the spectral window 1050–1770 cm^−1^ was used for analysis (Figure 4B). The EF was calculated from the characteristic HSA band at 1453 cm^−1^, assigned to the δ(CH_2_) scissoring deformation of amino acid side chains [68,69], according to:

$$EF=\frac{I_{SERS}/N_{SERS}}{I_{Raman}/N_{Raman}}$$

where I_SERS_ is the intensity of a specific analyte line in the SERS spectrum; N_SERS_ is the number of molecules that contribute to the SERS signal; I_Raman_ is the intensity of the same line in the normal (non-SERS) Raman spectrum measured under the same conditions; N_Raman_ is the number of molecules in the scattering volume for the normal scattering experiment. Identical droplet volumes (3 µL) and HSA concentrations were used for both substrates, ensuring comparable N.

The enhancement factor value calculated by comparing the amplitudes of the characteristic peak at 1453 cm^−1^ was approximately 9.6 × 10^3^, which corresponds to the literature values for self-assembled nanostructured silver films [39,61].

Taken together, the morphological consistency across batches, the acceptable spectral reproducibility, and the measured EF demonstrate that the fabricated silver substrates are suitable for the fungal polysaccharide markers SERS analysis.

### 3.2. Comparison of the SERS Spectra of Analyte Sets at a Fixed Concentration

**Figure 5:**
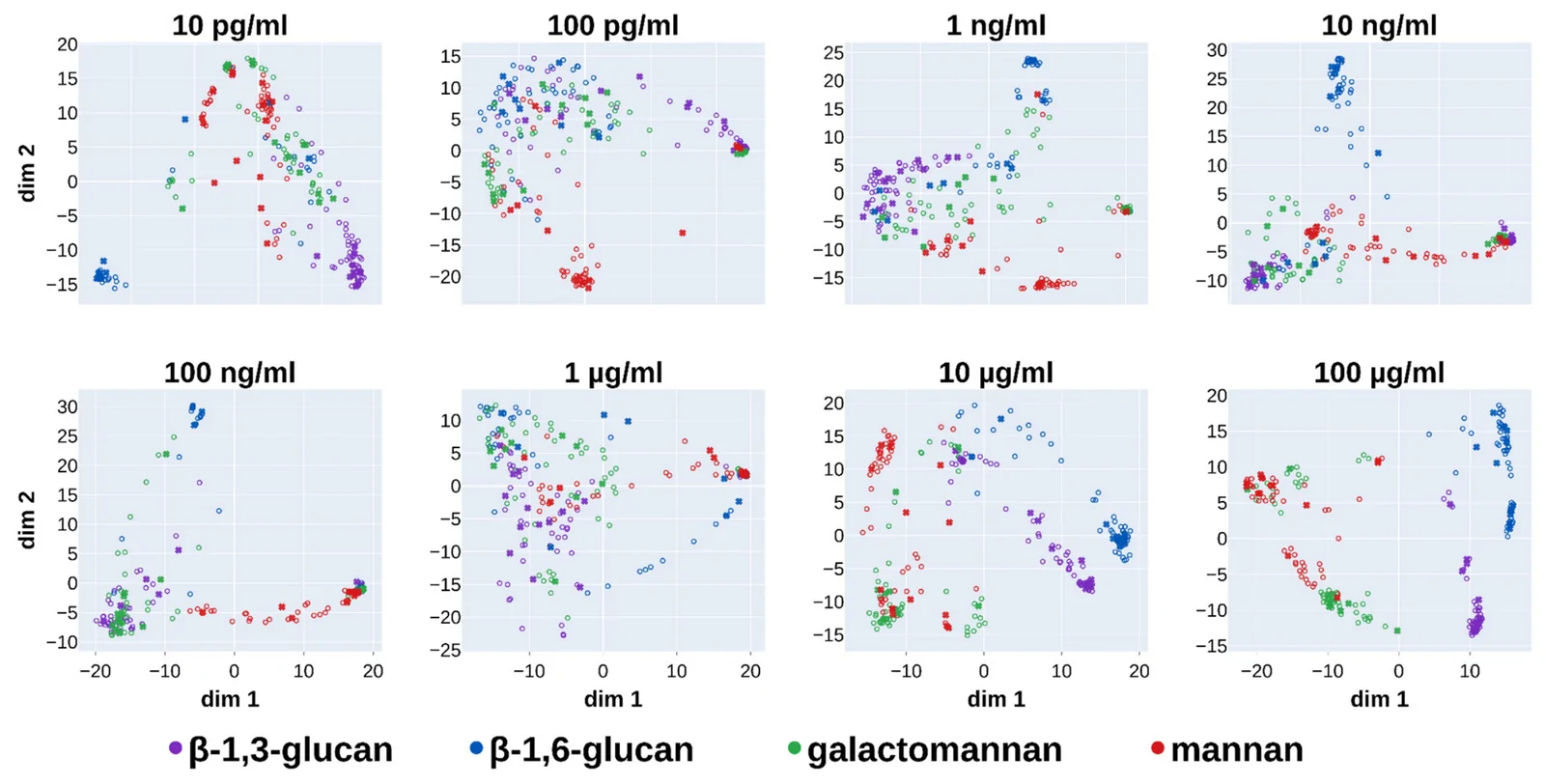
PCA score plots of the preprocessed analyte spectra at concentrations ranging from 10 pg/mL to 100 µg/mL. Each point represents a single spectrum. Color encodes the analyte class: β-1,3-glucan, β-1,6-glucan, mannan, and galactomannan. The circles indicate the training data, and the crosses indicate the test data. Separate panels correspond to different concentrations. The dim 1 and dim 2 axes are the first two principal components (directions of maximum variance). Point proximity reflects spectral similarity, and cloud separation indicates differences between the analytes.

After preprocessing, the measured SERS spectra were analyzed using machine learning methods. The first step involved unsupervised dimensionality reduction for visualization of the spectral datasets. Figure 5 shows scatter plots of the first two principal components for the SERS spectra of the analytes at a fixed concentration. Here, each point corresponds to one spectrum. The color/marker encodes the analyte class. Spatial proximity of points is interpreted as similarity of spectral profiles in multidimensional space after preprocessing. Cloud separation indicates the presence of persistent spectral differences. The corresponding PC1–PC2 loading plots for the PCA models are presented in the Supplementary Information (Figure S1). Each panel represents one concentration level and shows the Raman-shift variables that contribute most strongly to the variance explained by the first two principal components.

**Figure 6:**
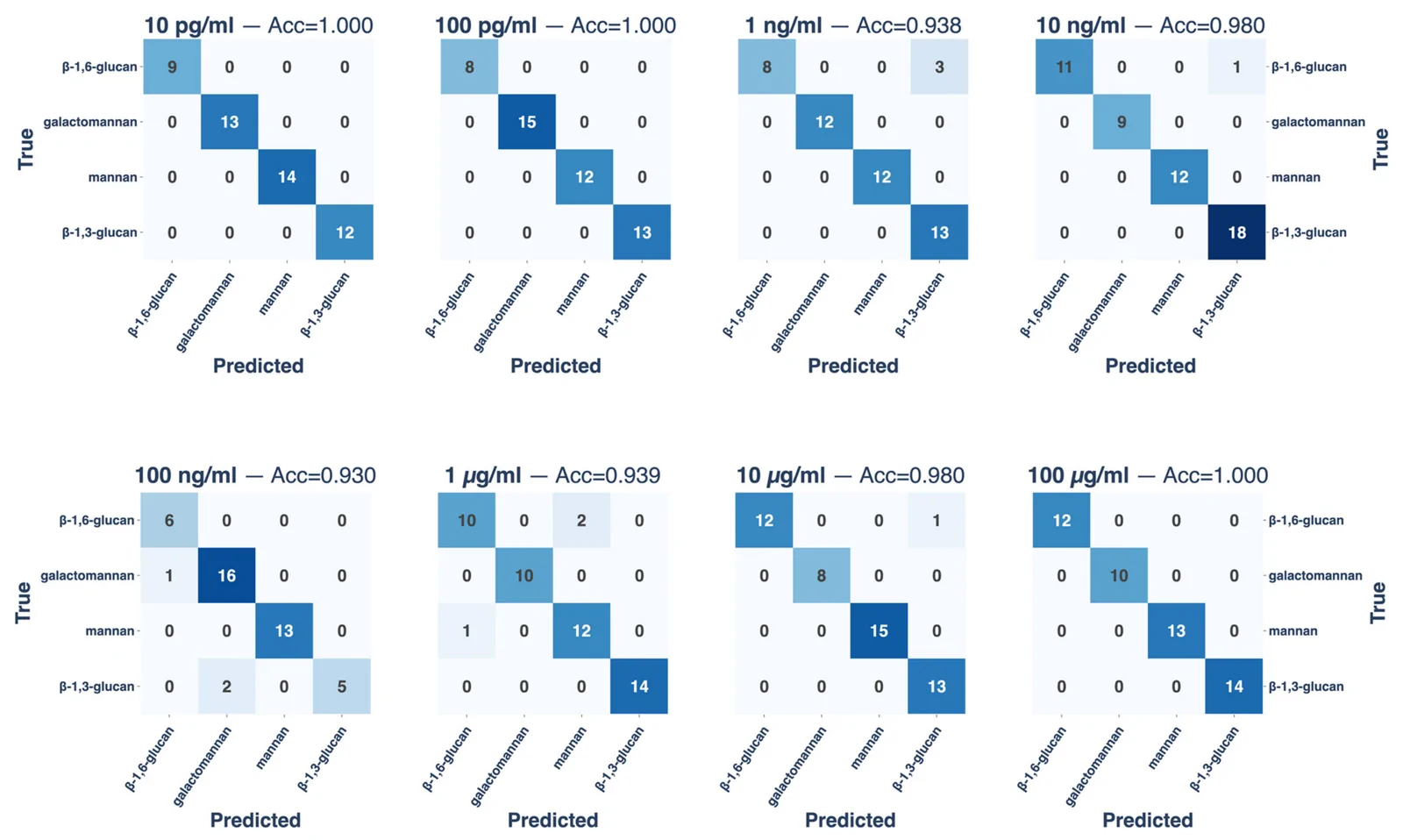
Confusion matrices showing classification accuracy for the analytes at concentrations from 10 pg/mL to 100 µg/mL. Diagonal values indicate correct classifications; off-diagonal values indicate misclassifications between analyte pairs.

It should be noted that the two-dimensional projections of the first two principal components reveal only a tendency for the spectral point clouds of different analytes to redistribute and do not in themselves constitute a classification decision. Nevertheless, this is already sufficient to investigate trends in spectral behavior as a function of molecular structure. Importantly, the shape of the point clouds clearly tends to shift depending on the concentration under consideration. In particular, the galactomannan of *A. fumigatus* sample most frequently exhibits the strongest separation from the other spectral point clouds, followed by β-1,3-glucan and β-1,6-glucan. The classification accuracy metrics are presented in Figure 6.

As seen in Figure 6, the metric matrices separate the spectra of all samples at each given concentration with very high accuracy. The lowest accuracy of 0.91 was observed at a concentration of 100 ng/mL, where the model confuses galactomannan and β-1,3-Glucan. The highest accuracy of 1.00 was obtained for both the lowest and the highest concentrations.

**Figure 7:**
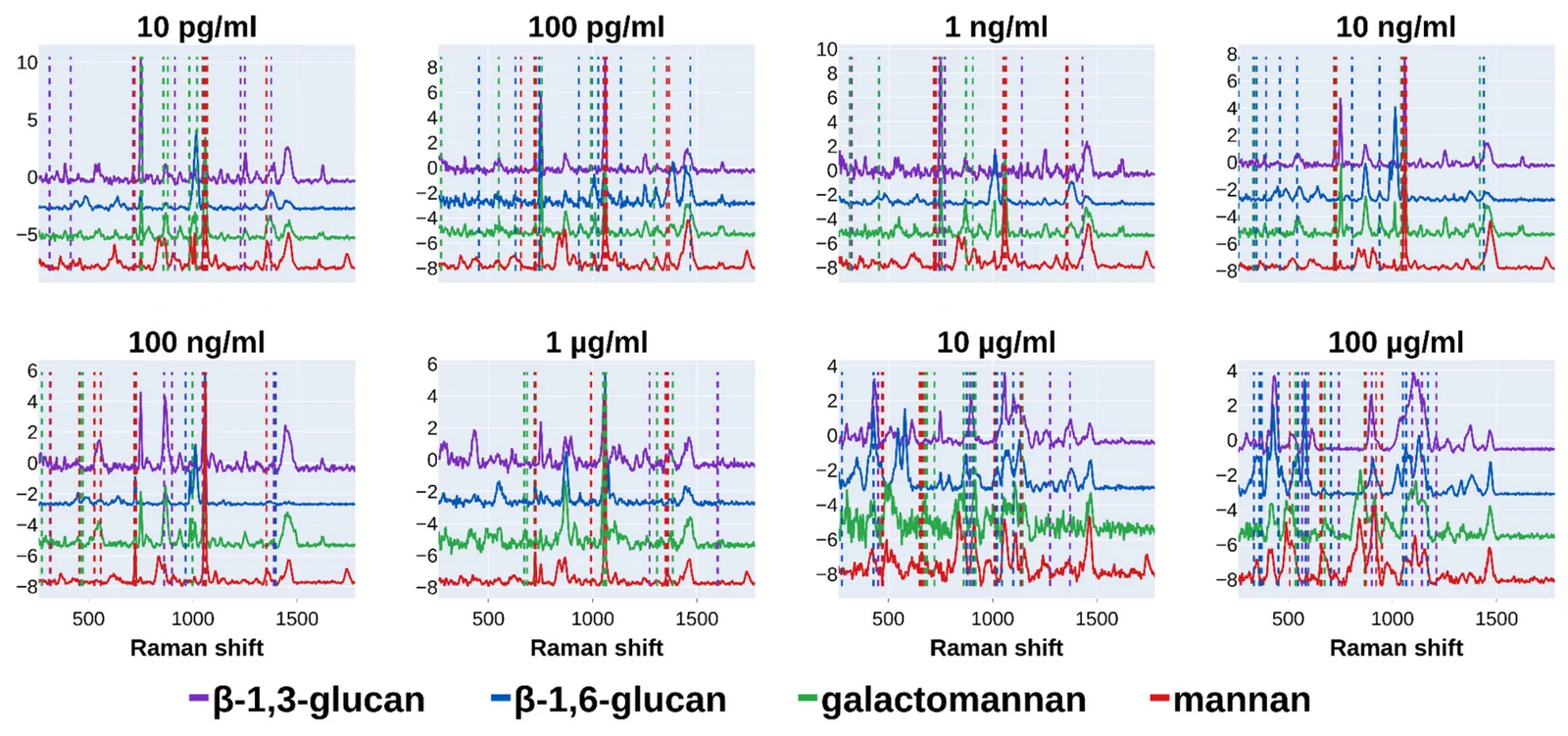
Spectral regions with the highest contribution to class separation (based on linear model coefficients). Solid lines show the averaged analyte spectra; dashed vertical lines mark the coordinates of the maximum absolute weights, interpreted as significant spectral loading features overlaid on the averaged spectra. The color of the loading lines matches that of the corresponding sample spectrum.

The vibrational bands responsible for the separation at each concentration were calculated as loadings and are presented in Figure 7. The positions of the vibrational bands corresponding to the identified loadings are presented in Table 1. The loadings are ranked in descending order of importance. Notably, a considerable number of spectral variables contribute to class separation, indirectly indicating marked spectral differences. Assignment of these vibrational bands requires further study.

### 3.3. Comparison of the SERS Spectra of a Set of Concentrations with a Fixed Analyte

**Figure 8:**
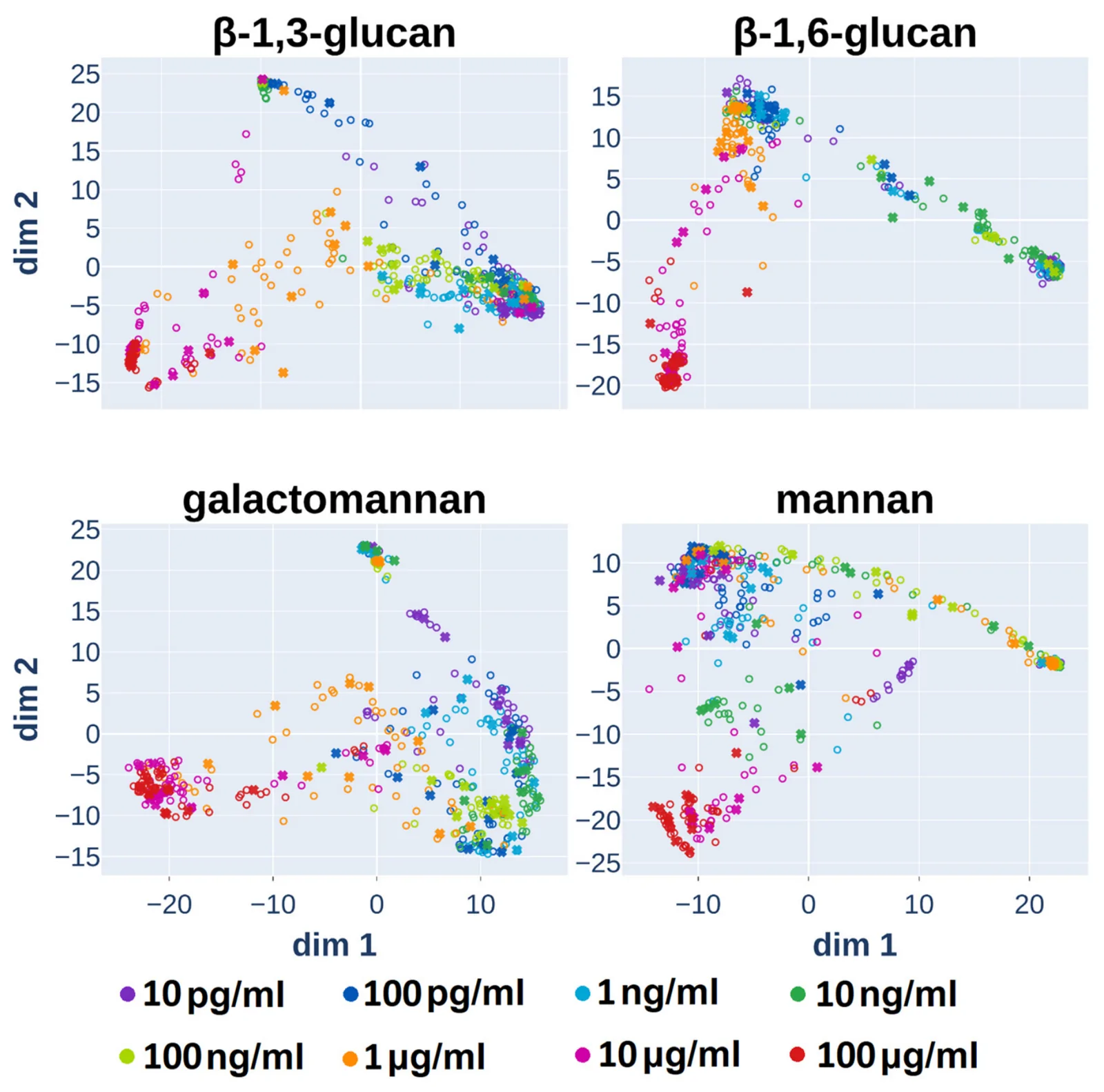
PCA score plots of analytes at different concentrations. Color encodes the analyte concentration from 10 pg/mL to 100 µg/mL. The circles indicate the training data, and the crosses indicate the test data. Separate panels correspond to different analytes. The dim 1 and dim 2 axes are the first two principal components (directions of maximum variance).

To assess the spectral changes of the investigated polysaccharides upon concentration variation in aqueous solution, their SERS spectra were processed according to Section 2.6.2. The corresponding PCA scores plots are presented in Figure 8. The corresponding PC1–PC2 loading plots for the PCA models are presented in the Supplementary Information (Figure S2).

The projections of the spectral point clouds onto two components demonstrate behavior similar to that observed when comparing the polysaccharides with one another at a fixed concentration, appearing as diffuse point clouds. It should be noted that this result indicates a trend but does not constitute an exact representation of classification, since it shows projections onto only the first two principal component axes.

Each sample shows a tendency to separate relatively high concentrations of 10–100 μg/mL. In contrast, starting from 1 µg/mL and less, differences begin to appear. Overall, a change in the spectrum is observed for all samples with a decrease in concentration after 1 µg/mL. This was explained in earlier work by the influence of the substrate electrostatic field [39]. The concentration of 1 µg/mL is transitional: for β-1,3-glucan, β-1,6-glucan, and galactomannan, their spectral point cloud is located rather between the point clouds of higher concentrations. However, for mannan, the point cloud tends to increase in area and overlap with the point clouds of lower concentrations. Additionally, for β-1,3-glucan, β-1,6-glucan, and galactomannan, a gradual shift of the point clouds from higher to lower concentrations is observed.

**Figure 9:**
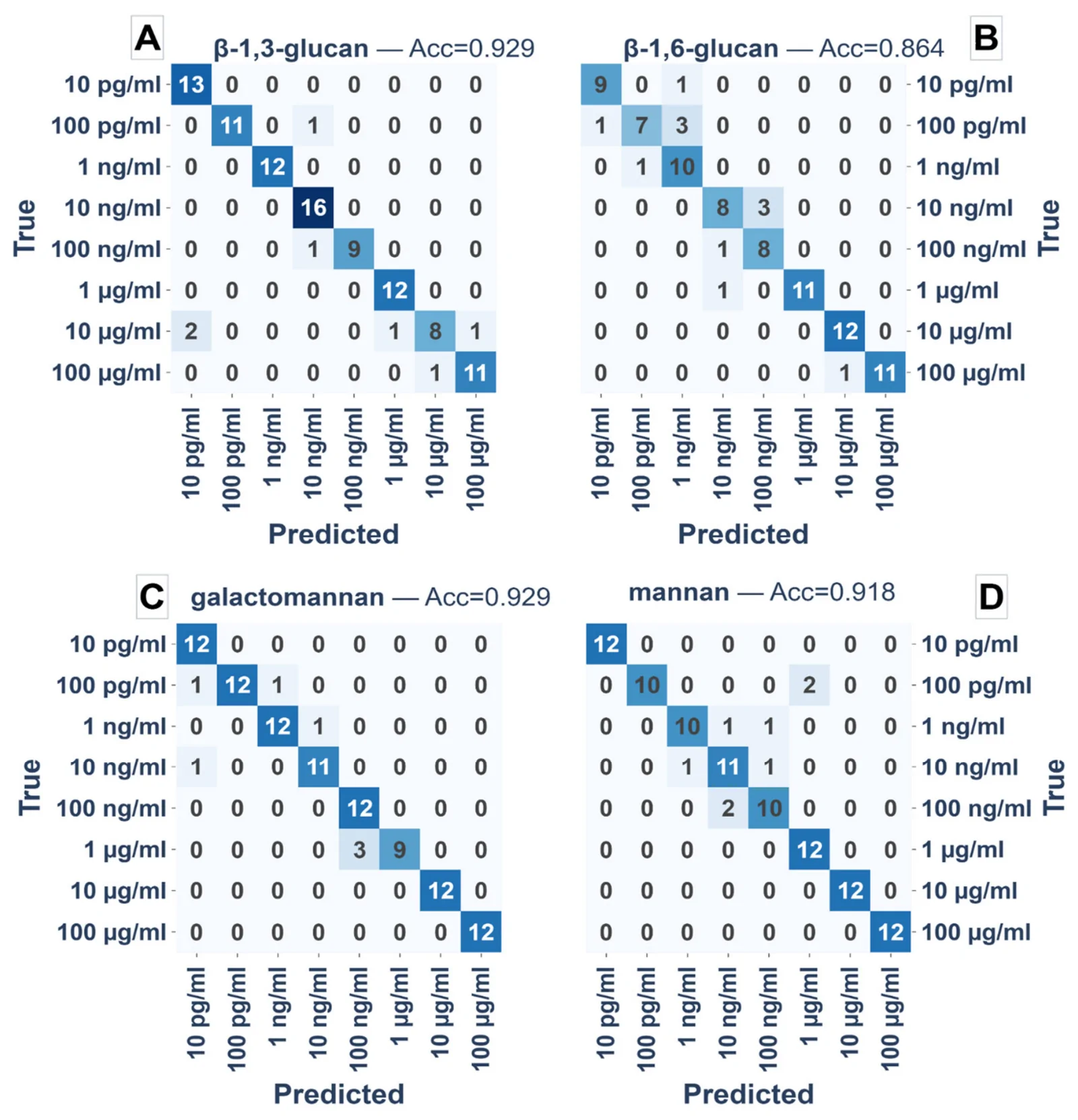
Classification accuracy matrices for the concentration-series separation of individual analytes: (**A**) β-1,3-glucan; (**B**) β-1,6-glucan; (**C**) galactomannan; (**D**) mannan. The numerical values on the diagonal elements correspond to correct predictions; the off-diagonal elements represent misclassifications (confusions) between pairs of analytes.

Of course, this is not a pronounced tendency for a representation involving projections onto only two axes, but it is sufficient to expect spectral separation in classification methods, as will be shown below by the metric matrices in Figure 9.

As seen in Figure 9, the classification accuracy matrices for all analytes across concentrations show high classification accuracies of 0.84–0.96 for all samples. The galactomannan and mannan samples are distinguished most accurately, with accuracies of 0.95 and 0.96. β-1,6-glucan and β-1,3-glucan are separated somewhat less accurately. Only neighboring concentrations are slightly confused, for example, 10 and 100 ng/mL.

**Figure 10:**
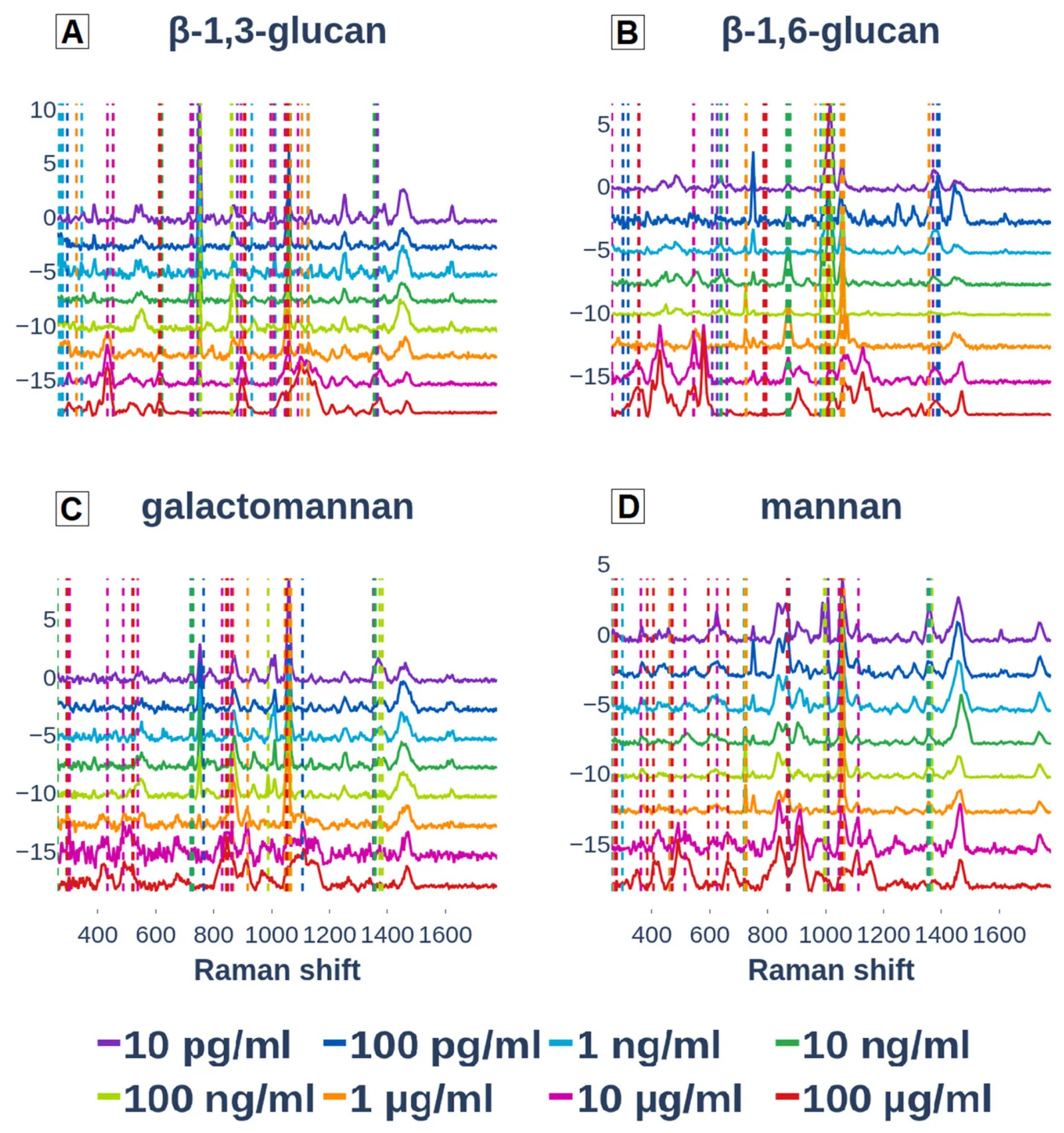
Dashed vertical lines on the averaged spectra indicate the loading positions for each concentration of the respective sample: (**A**) β-1,3-glucan; (**B**) β-1,6-glucan; (**C**) galactomannan; (**D**) mannan. The color of each loading line corresponds to that of the spectrum at the respective concentration.

To identify the vibrational bands in the spectra where separation occurs, loadings were constructed showing which specific peaks contribute to the separation. The loadings are shown in Figure 10. The frequencies corresponding to the determined loads are given in Table 2.

The loadings are listed in descending order of significance. It should be noted that a considerable number of spectral features contribute to the separation. This indirectly indicates substantial differences between the spectra. Assignment of the vibrational bands requires further investigation.

### 3.4. Calibration of Analytes in the HSA Model Matrix

Calibration construction for the HSA model solutions was formulated as a regression problem: the logarithm of concentration y = log_10_(C) was predicted from the spectral vector X. As the regression model, Partial Least Squares with L1 regularization (PLS-L1) was employed; this approach simultaneously accounts for the correlated structure of spectral features and performs sparse feature selection.

Before training, the features were standardized (Standard Scaler), ensuring a comparable scale for the spectral readings. Generalization performance was evaluated on independent training and test sets, enabling an unbiased assessment of calibration transferability.

For quantitative evaluation, the coefficient of determination R^2^ and mean squared error (MSE) were used on the logarithmic concentration scale.

Spectrum-level metrics were reported for each polysaccharide, and specimen-level metrics were calculated from the medians of accepted spectra for the eight independently prepared blind specimens.

**Figure 11:**
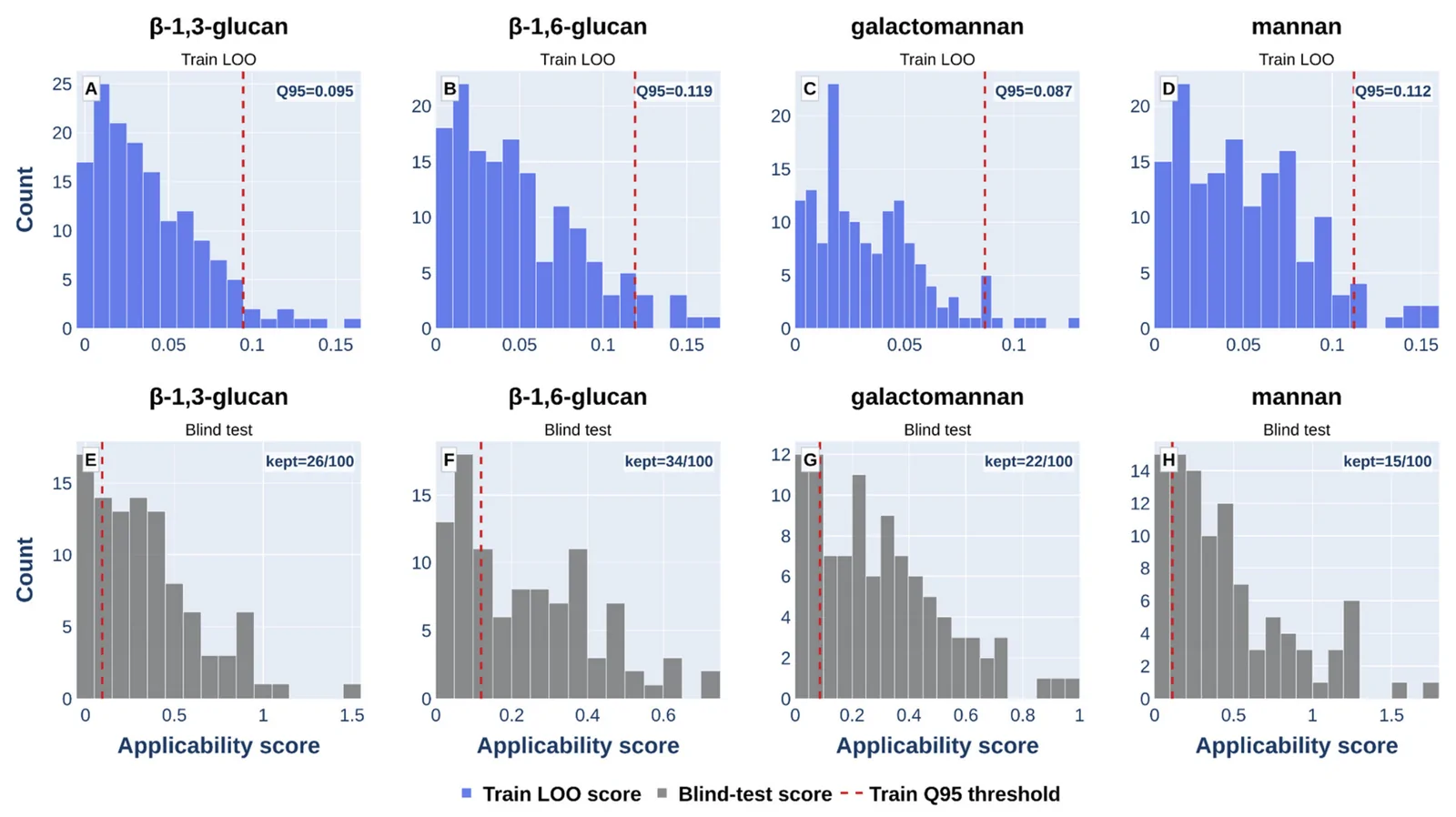
Distributions of the train-derived spectral-trajectory applicability score for the PLS-L1 calibration. Training leave-one-out distributions are shown for (**A**) β-1,3-glucan, (**B**) β-1,6-glucan, (**C**) galactomannan, and (**D**) mannan; blind-test distributions are shown for (**E**) β-1,3-glucan, (**F**) β-1,6-glucan, (**G**) galactomannan, and (**H**) mannan.

Figure 11 presents the distributions of the train-derived applicability score *a_i* and should be interpreted as a visualization of the applicability-domain decision rather than as a calibration plot. Panels A–D show leave-one-out scores for the training spectra, whereas panels E–H show the corresponding scores for the blind spectra. The red dashed line in each panel denotes the 95th percentile of the training distribution used as the acceptance threshold. The thresholds were 0.095, 0.119, 0.087, and 0.112 log10 units for β-1,3-glucan, β-1,6-glucan, galactomannan, and mannan, respectively.

The broader right tails of the blind-test distributions correspond to spectra for which the PLS-L1 prediction was inconsistent with the position of the spectrum relative to the training concentration trajectory. The criterion retained 26, 34, 22, and 15 out of 100 blind spectra for β-1,3-glucan, β-1,6-glucan, galactomannan, and mannan, respectively.

**Figure 12:**
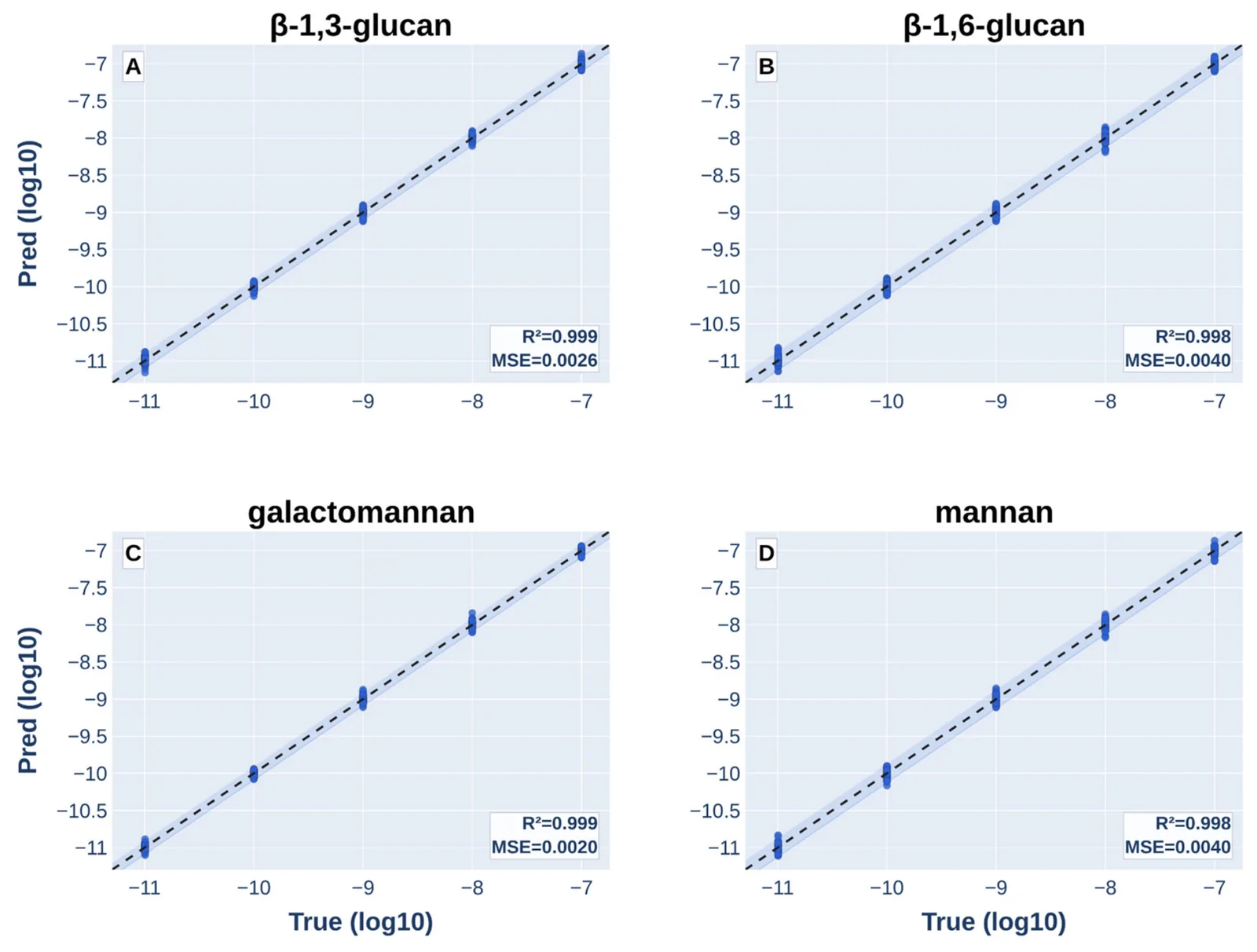
PLS-L1 calibration plots for the training set: (**A**) β-1,3-glucan; (**B**) β-1,6-glucan; (**C**) galactomannan; (**D**) mannan. True and predicted concentration values are presented on a logarithmic scale of y = log_10_(C + ε). The dashed diagonal line corresponds to ideal prediction; the band around the diagonal represents the typical spread of model errors.

The result of applying the described procedure to the training set is shown in Figure 12, which presents the calibration plots obtained under cross-validation. The points exhibit almost perfect agreement with the diagonal corresponding to ideal prediction (R^2^ ≈ 1.00), indicating that the PLS-L1 model correctly recovers the relationship between the spectral profile and concentration on the training data.

The threshold values define the acceptable deviation bounds determined from the error distribution on the training set. This criterion is then transferred to the test data: only spectra whose model error falls within the typical range observed during training are retained. Concentrations of 150 pg/mL and 500 pg/mL were selected as the test concentrations. These concentrations are representative of the physiologically relevant concentration range of the investigated polysaccharides and were therefore considered suitable for evaluating the performance of the model across the clinically relevant concentration range.

**Figure 13:**
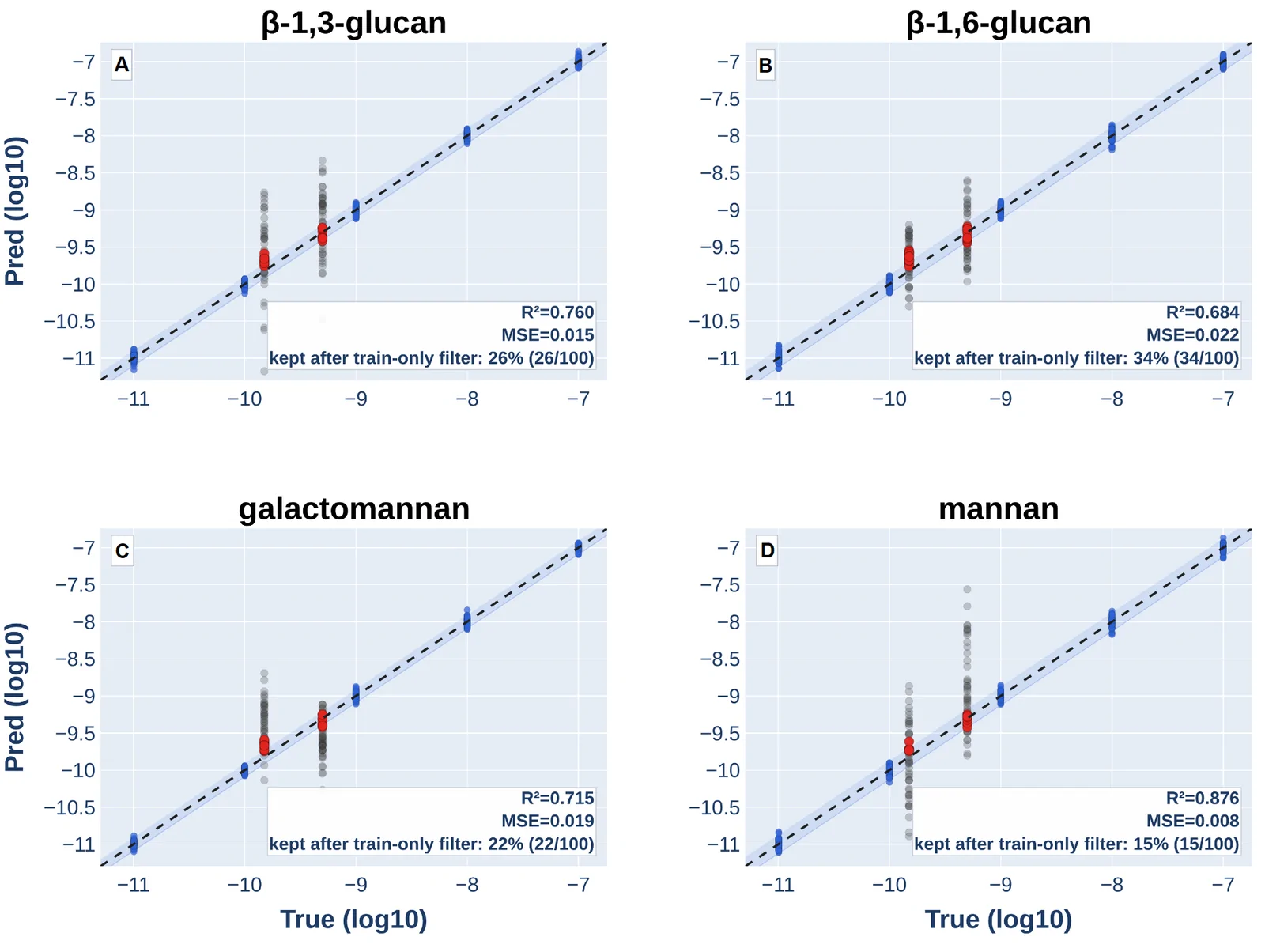
PLS-L1 blind-test calibration in the HSA model matrix for (**A**) β-1,3-glucan, (**B**) β-1,6-glucan, (**C**) galactomannan, and (**D**) mannan. Blue points represent the training set, gray points represent all blind spectra before applicability filtering, and red points represent blind spectra accepted by the train-derived spectral-trajectory criterion. The black dashed diagonal denotes ideal agreement, and the shaded band represents ±2 standard deviations of the training residuals. R^2^ and MSE are calculated for the accepted blind spectra. The measured blind concentrations were used only for post-prediction validation and not for the acceptance decision.

Figure 13 presents the blind-test results obtained with the train-only applicability criterion. Blue points show the training-set predictions, gray points show all blind-spectrum predictions before filtering, and red points show blind spectra accepted by the spectral-trajectory criterion. The acceptance decision was fixed before the measured blind concentration was introduced for validation.

At the spectrum level, the accepted subsets yielded R^2^ values of 0.760, 0.684, 0.715, and 0.876 and MSE values of 0.015, 0.022, 0.019, and 0.008 log10^2^ units for β-1,3-glucan, β-1,6-glucan, galactomannan, and mannan, respectively. At the specimen level, the medians of the accepted spectra for the eight independently prepared blind specimens yielded R^2^ = 0.834 and MSE = 0.011 log10^2^ units.

The spectral positions of the loadings used to construct the calibration curves are given in Table 3.

Analysis of Table 3 reveals a large number of vibrational bands responsible for the separation of the spectra. This indirectly indicates substantial modification of the matrix upon introduction of such minute quantities of the investigated analytes. It should also be noted that the vibrational bands overlap with those listed in Table 2, which present the loadings responsible for the separation of pure analytes spectra at different concentrations (for β-1,3-glucan: 410, 567, 650, 1608, 410, 1682, 1442, 310, 809, 1600, 584, 1091 cm^−1^; for β-1,6-glucan: 497, 1022, 965, 572, 892, 292, 406, 572, 892, 1102 cm^−1^; for galactomannan: 721, 723, 725, 726, 728, 332, 262, 1039, 862, 933, 894, 1458 cm^−1^; for mannan: 1352, 173, 559, 555, 1353, 948, 1460, 949, 697, 818, 87, 1115 cm^−1^). The incomplete overlap across different concentrations indicates that in the matrix-based experiment, we observe an integrative change in the overall spectrum of both the matrix and the introduced substance.

Certainly, the proposed matrix model does not represent a fully realistic clinical matrix. It was selected as a model system for developing the mathematical framework capable of detecting low concentrations of the introduced polysaccharides, which serve as markers of fungal infections, against a high-molecular-weight background matrix. However, since albumin is known to be the predominant protein component of blood serum in the molecular-weight range below 100 kDa, it can be considered the principal matrix component against which fungal infection marker polysaccharides are to be detected in the proposed model. This molecular-weight range can be achieved relatively easily using centrifugal filters during plasma sample preparation in combination with routine clinical purification procedures.

In future studies, the proposed mathematical framework will be transferred to real clinical samples, taking into account the clinical variability of the matrix. This variability will be assessed using samples from both patients and conditionally healthy donors. The identified vibrational bands characteristic of the respective polysaccharides will also be used for analysis of diverse clinical matrices.

## 4. Conclusions

This study demonstrates the feasibility of analyzing the polysaccharide components of pathogenic fungal cell walls—β-1,3- and β-1,3-D-glucan, as well as *C. albicans* mannan and *A. fumigatus* galactomannan—by SERS combined with machine learning. A wide concentration range from 10 pg/mL to 100 µg/mL was investigated for aqueous solutions. Concentration-dependent spectral profile dynamics were demonstrated for each sample, enabling their determination in model biological media within the clinically significant concentration range. Solutions of human serum albumin at a physiological concentration of 45 mg/mL was used to simulate model blood serum. The investigated samples were examined over a concentration range from 100 ng/mL to 10 pg/mL with tenfold dilution steps.

Calibration was formulated as a regression problem using PLS-L1 model and was shown to correctly recover the relationship between the spectral profile and concentration on the training data. Robust z-score filtering applied to the PLS predictions removed atypical and noisy model predictions, thereby improving calibration accuracy. R^2^ for the calibration model of each investigated sample was approximately 1.00, corresponding to near-ideal model prediction on the training data. To validate the calibration model, additional solutions of all samples at concentrations of 150 and 500 pg/mL were prepared in spiked HSA solution and used as test data for blind testing. The combined use of regularized PLS regression and robust z-score filtering for the test spiked solutions yielded calibration metrics in the range of R^2^ = 0.85–0.87 at MSE ≈ 0.01.

The described study demonstrated the feasibility of using the SERS method for the qualitative determination of markers of fungal cell walls and, in the future, can be expanded to polysaccharide markers of other types of pathogens. Thus, the resulting combination of spectral analysis and machine learning methods could form the basis for the development of a clinical method for the quantitative determination of polysaccharides.

## Figures and Tables

**Table 1:** The positions of the vibrational bands corresponding to the Raman-shift positions associated with the largest absolute linear-model coefficients when comparing polysaccharides in different concentrations.

	β-1,6-Glucan	β-1,3-Glucan	Mannan	Galactomannan
10 pg/mL	1048, 719, 1058, 1051, 1050, 1060, 1062, 1057, 1063, 1055	1376, 1249, 312, 414, 1229, 713, 914, 758, 749, 717	1051, 1353, 1067, 719, 1050, 1060, 1062, 1057, 1063, 1055	1020, 983, 858, 880, 1069, 749, 1067, 758, 1057, 1063
100 pg/mL	455, 745, 629, 995, 1027, 1136, 1468, 997, 743, 933	1053, 1063, 721, 1051, 656, 1067, 723, 1355, 1057, 1055	1366, 1053, 1067, 656, 726, 1355, 1057, 721, 723, 1055	728, 549, 1294, 992, 756, 272, 1355, 1063, 1055, 1057
1 ng/mL	728, 1053, 1057, 1358, 1051, 726, 1055, 1063, 719	771, 1141, 324, 314, 750, 455, 1431, 317, 743, 745	1355, 1060, 1063, 1358, 1053, 1051, 1055, 726, 719	873, 905, 316, 455, 719, 756, 728, 1057, 721, 1063
10 ng/mL	262, 336, 348, 394, 460, 542, 805, 939, 1418, 1438	719, 721, 726, 728, 730, 1048, 1050, 1053	721, 726, 728, 1048, 1051, 1053, 1065, 1063	330, 1418, 1041, 1051, 721, 730, 719, 1065, 723, 728
100 ng/mL	1396, 999, 965, 1397, 472, 728, 725, 721, 1063, 723	862, 1388, 314, 1062, 1048, 900, 726, 1063, 1053, 721	455, 721, 528, 318, 559, 1353, 1061, 726, 719, 1053	276, 472, 728, 461, 999, 559, 1063, 725, 723, 721
1 μg/mL	992, 1060, 1358, 723, 1360, 1357, 726, 1063, 725, 721	1057, 1060, 1599, 723, 1361, 1272, 1357, 1600, 1058, 723	1350, 992, 994, 1358, 1360, 1355, 726, 723, 1357, 724	1309, 685, 672, 1050, 1053, 1055, 1384, 106, 721, 726
10 μg/mL	876, 1020, 1009, 1044, 910, 429, 276, 1134, 1100, 1022	276, 1276, 901, 451, 891, 1100, 1371, 1015, 429, 1136	474, 1009, 665, 470, 1138, 652, 661, 657, 650, 654	862, 474, 917, 685, 721, 1144, 657, 676, 901, 661
100 μg/mL	1050, 704, 1170, 372, 453, 545, 1065, 334, 362, 584	566, 743, 1141, 901, 597, 334, 545, 1211, 1095, 1065	661, 507, 657, 949, 921, 873, 871, 656, 654, 659	674, 867, 538, 507, 656, 536, 654, 661, 659, 921

**Table 2:** Spectral positions of loads when comparing different concentrations within each sample.

Concentration	β-1,6-Glucan	β-1,3-Glucan	Mannan	Galactomannan
10 pg/mL	1371, 497, 631, 1388, 455, 939, 1370, 637, 1016, 1022	567, 1608, 1415, 384, 1360, 410, 278, 941, 979, 650	1352, 1102, 1067, 1370, 623, 1051, 1053, 1065, 1063, 725	778, 492, 631, 1055, 1352, 1353, 1357, 1355, 725, 723
100 pg/mL	1043, 441, 623, 1155, 1392, 1386, 644, 637, 538, 1018	476, 1032, 715, 758, 1355, 1063, 1065, 728, 725, 723	1063, 1376, 555, 1357, 1730, 459, 404, 721, 462, 725	1389, 723, 1063, 276, 1430, 427, 1065, 1067, 728, 725
1 ng/mL	455, 642, 633, 320, 468, 1015, 726, 1018, 1016, 1022	1086, 1083, 410, 1422, 1286, 976, 312, 1277, 1608, 1392	298, 1102, 534, 1734, 726, 559, 723, 1740, 719, 542	1355, 723, 728, 1060, 1063, 1062, 725, 1067, 721, 1065
10 ng/mL	—	1682, 798, 1177, 725, 1360, 1065, 1355, 723, 1063, 726	1389, 605, 1355, 340, 1353, 723, 726, 1053, 725, 721	474, 1355, 1055, 507, 1357, 721, 1492, 725, 723, 728
100 ng/mL	1358, 1063, 990, 992, 1058, 965, 1060, 1357, 725, 726	942, 1442, 880, 756, 310, 667, 754, 1379, 860, 862	352, 948, 1740, 542, 726, 1051, 721, 1053, 719, 723	1002, 805, 1384, 1428, 723, 296, 745, 726, 1053, 1055
1 μg/mL	1053, 1060, 1058, 1355, 1358, 965, 1357, 1063, 725, 726	1386, 809, 1127, 1168, 1600, 1032, 515, 1058, 326, 678	1460, 728, 726, 1076, 1357, 404, 1069, 462, 1063, 725	1217, 606, 665, 1039, 1428, 1294, 680, 1055, 578, 332
10 μg/mL	1422, 348, 1276, 354, 1043, 1330, 892, 545, 572, 433	917, 1296, 726, 1046, 763, 439, 1352, 1141, 614, 526	1350, 949, 805, 1025, 1585, 765, 772, 459, 505, 802	262, 831, 862, 648, 1194, 815, 392, 933, 640, 466
100 μg/mL	1239, 406, 292, 336, 1324, 1102, 370, 892, 572, 433	1151, 584, 1067, 1773, 901, 439, 1091, 612, 1124, 614	1143, 1136, 1294, 697, 278, 1115, 818, 1131, 871, 384	1458, 816, 933, 642, 894, 429, 1194, 292, 1034, 646

**Table 3:** Spectral positions of loadings responsible for the discrimination of spectra in the HSA model matrix.

HSA + β-1,3-Glc	302, 958, 1444, 412, 1546, 1683, 1013, 394, 1602, 1711, 1002, 561, 1696, 1023, 421, 802, 350, 1516, 1074, 260, 1476, 358, 1269, 1345, 646, 1625, 1089, 565, 584, 1490
HSA + β-1,6-Glc	1029, 894, 608, 1168, 972, 493, 589, 296, 815, 1294, 1103, 892, 587, 282, 1143, 495, 825, 408, 854, 1306, 710, 1006, 965, 476, 572, 1077, 1759, 1289, 1316, 978
HSA + Ag. A.f	1016, 1637, 284, 260, 957, 1458, 1178, 725, 787, 793, 858, 1036, 1279, 1767, 1081, 964, 1466, 899, 1247, 1648, 1182, 1460, 1450, 953, 937, 865, 942, 730, 990, 338
HSA + Ag. C.a	926, 1006, 867, 1112, 813, 559, 656, 946, 1247, 1642, 1348, 793, 901, 1479, 280, 882, 654, 1727, 987, 495, 1381, 1452, 627, 1216, 593, 706, 591, 687, 695, 1002

## Data Availability

All data generated or analyzed during this study are included in this published article. Any additional data will be available upon request.

## Supplementary Materials

The following supporting information can be downloaded at: https://www.mdpi.com/article/10.3390/bios16090502/s1, Figure S1: PCA loading spectra corresponding to the fixed-concentration score plots; Figure S2: PCA loading spectra for the concentration-series models PC1/PC2 loadings with vertically offset concentration mean spectra.
