# Supplementary material for: Detection of Polysaccharide Markers of Fungal Infections by Surface-Enhanced Raman Scattering and Machine Learning Methods
Source: Biosensors (Basel). 2026 Sep 8;16(9):502. doi: 10.3390/bios16090502 (PMC13604236; doi:10.3390/bios16090502)
Supplement: Supplementary file 1 [file biosensors-16-00502-s001.zip › biosensors-4498119-supplementary.pdf]

biosensors

Article

Detection of polysaccharide markers of fungal infections by surface-enhanced Raman scattering and machine learning methods

Julia Yu. Zvyagina[1,*], Robert R. Safiullin[1,2], Andrey S. Naboko[1], Victor I. Polozov[1], Irina A. Boginskaya[1,*], Marina V. Sedova[1], Vadim B. Krylov[3], Dmitry V. Yashunsky[3], Dmitry A. Argunov[3], Nikolay E. Nifantiev[3,*], Ilya A. Ryzhikov[1,4], Alexander M. Merzlikin[1] and Andrey N. Lagarkov[1]

Supplementary materials

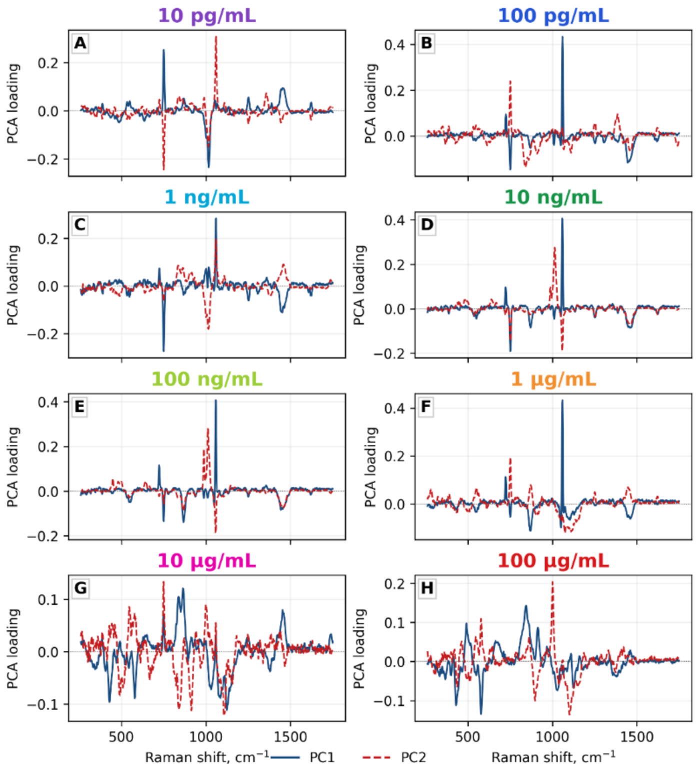

Figure S1. PCA loading spectra corresponding to the fixed-concentration score plots.

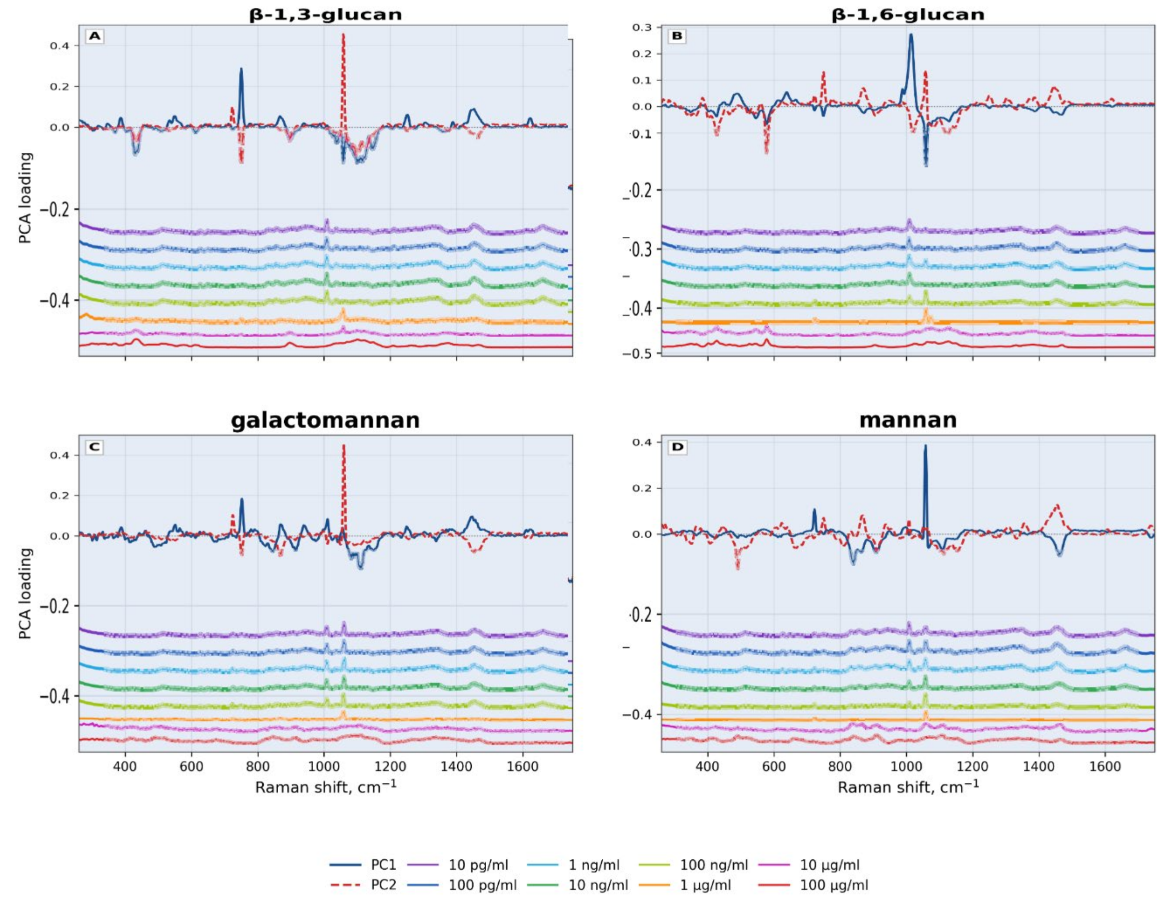

Figure S2. PCA loading spectra for the concentration-series models PC1/PC2 loadings with vertically offset concentration mean spectra.
